# Transcriptome dynamics-based operon prediction in prokaryotes

**DOI:** 10.1186/1471-2105-15-145

**Published:** 2014-05-16

**Authors:** Vittorio Fortino, Olli-Pekka Smolander, Petri Auvinen, Roberto Tagliaferri, Dario Greco

**Affiliations:** 1 Department of Computer Science (DI), NeuRoNe Lab, University of Salerno, via ponte don Melillo 84084, Fisciano, (SA), Italy; 2 Department of Pharmaceutical and Biomedical Sciences (FARMABIOMED), University of Salerno, via ponte don Melillo, Fisciano, (SA) 84084, Italy; 3 Unit of Systems Toxicology, Finnish Institute of Occupational Health (FIOH), Topeliuksenkatu 41b, Helsinki 00250, Finland; 4 Institute of Biotechnology, University of Helsinki, Helsinki, Finland

**Keywords:** Operons, Computational prediction, Condition-dependent operon maps, RNA-seq data analysis

## Abstract

**Background:**

Inferring operon maps is crucial to understanding the regulatory networks of prokaryotic genomes. Recently, RNA-seq based transcriptome studies revealed that in many bacterial species the operon structure vary with the change of environmental conditions. Therefore, new computational solutions that use both static and dynamic data are necessary to create condition specific operon predictions.

**Results:**

In this work, we propose a novel classification method that integrates RNA-seq based transcriptome profiles with genomic sequence features to accurately identify the operons that are expressed under a measured condition. The classifiers are trained on a small set of confirmed operons and then used to classify the remaining gene pairs of the organism studied. Finally, by linking consecutive gene pairs classified as operons, our computational approach produces condition-dependent operon maps. We evaluated our approach on various RNA-seq expression profiles of the bacteria *Haemophilus somni*, *Porphyromonas gingivalis, Escherichia coli and Salmonella enterica*. Our results demonstrate that, using features depending on both transcriptome dynamics and genome sequence characteristics, we can identify operon pairs with high accuracy. Moreover, the combination of DNA sequence and expression data results in more accurate predictions than each one alone.

**Conclusion:**

We present a computational strategy for the comprehensive analysis of condition-dependent operon maps in prokaryotes. Our method can be used to generate condition specific operon maps of many bacterial organisms for which high-resolution transcriptome data is available.

## Background

Prokaryotic operons are sets of genes encoded on the same strand of DNA that are co-transcribed and are often identified by the presence of promoters and terminators [1]. Genes transcribed in a single operon are functionally related and make up a part of a metabolic pathway [2,3]. Therefore, the identification of genes that are grouped together into operons is a key step toward the reconstruction of complex regulatory networks. However, the mechanisms of operon formation are still poorly understood and experimental methods for genome-wide identification of operon structures are laborious [4]. For this reason, developing computational strategies to effectively predict operons has become an important issue. In the past decade, many computational methods for predicting operons were developed based on DNA sequence features. These, for instance, include algorithms that rely on the intergenic distance [5,6], the gene cluster conservation [3,7] and the function commonality [8]. Furthermore, operon prediction can be enhanced by considering multiple genomic properties [9] or combining computational methods with experimental gene expression data [10]. Most of the existing methods predict operons using models trained on a set of experimentally defined operons [11] and, consequently, they are limited to work only with well characterized genomes, such as *E. coli* and *B. subtilis*[8]. Moreover, these methods tend to define just a single, ‘optimal’ operon map attempting to group genes that would be expressed together regardless of the experimental conditions. This is in disagreement with recent RNA-seq based transcriptome studies showing frequent condition-dependent changes of the expression patterns as well as modifications of the operon structure both in bacteria and archaea [12,13]. Using tiling arrays and RNA-seq data, changes in the operon structures in response to different experimental conditions have been observed and novel operons have been described [14,15].

These findings challenge the definition of operon structure and make debatable the approaches used by the current methods. It is in fact evident that the operons are ‘dynamic’ structures, *i.e.* they are able to produce different transcriptional units (TUs), poly- and mono-cistronic mRNAs, depending on the environmental or growth conditions of the cell [12]. In such context, it becomes important to develop computational methods to identify different operon maps for different environmental conditions.

In this study, we propose a computational method to produce highly accurate condition-dependent operon maps by integrating dynamic RNA-seq data with static DNA-sequence based information. The proposed computational method was implemented in R (Additional file 1).

## Methods

### Strategy overview

**Figure 1:**
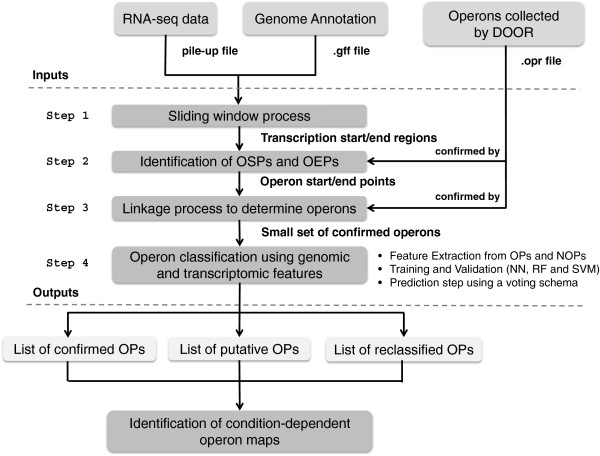
**Schematic overview of the method.** The inputs are: a whole-transcriptome RNA-seq profile of a prokaryotic organism, the genome annotations and the corresponding map of operons collected by DOOR. The core process is represented by four steps. The first three steps determine a set of confirmed operons. While, in the last step the system trains and validates a NN-, a RF- and a SVM-based classifier on a list of confirmed OPs and NOPs. In output, these classifiers are used to reassess the operon structure annotated in DOOR. Further, a validation process is accomplished to computationally verify that there are not predicted regulatory signals between adjacent genes indicated as operon pairs.

In order to avoid confusion with the terms operon and TU in this study, we use only the term ‘operon’ to indicate a set of genes that are transcribed as a unit under a certain condition [16]. Therefore, the prediction task that we considered here is the following: given the genome sequence and a RNA-seq based transcriptome profile we wanted to predict all the operons that are expressed under a measured condition. We achieved this by combining static (e.g., DNA sequence properties) and dynamic data sources (e.g., RNA-seq data) in order to define classifiers that can correctly assign genes to operons and to identify new potential operons. Since the transcriptome of an organism is dynamic and condition dependent, we utilized the RNA-seq mapped reads to determine the transcription start/end points. Then, we extracted static (*e.g.*, intergenic distance) and dynamic (*e.g.*, transcription level of intergenic regions) features from this small set of operons to train/validate three classification models: Random Forest (RF), Neural Network (NN) and Support Vector Machine (SVM). Figure 1 illustrates the synthetic scheme of the proposed solution to build condition-dependent operon maps.

The key elements of our approach are i) to identify the start/end transcription points and the expression levels of annotated genes and intergenic regions, ii) to determine a set of confirmed operons using DOOR [17] annotations iii), to use both genomic and transcriptomic features in order to train and validate models for the classification of known operon pairs (positive class) and non-operon pairs (negative class), and iv) to use these trained models to classify unlabeled gene pairs. Since the genes within an operon are co-transcribed, no regulatory signals should be present between the genes of the same operon pairs. Therefore, we used the predicted promoters and terminators to increase reliability (Figure 1).

### Determination of transcript boundaries and expression levels for the annotations

In order to specify the location of active transcription units in the genome, we first estimated the coverage depth of reads mapped per nucleotide/base using pileup files. Second, we used a sliding window correlation procedure to identify regions surrounding putative transcription start- and end-sites. The expression values for each gene and intergenic region were calculated using the RPKM method [18,19].

The first input is a pileup file corresponding to a single transcriptome RNA-seq profile (Figure 1). This represents the genome-wide signal map in which the alignment results are represented in a per-base format. We exploited this file to compute the coverage depth, which is the number of reads mapped for each genomic position, and determine the putative transcription start- and end-site positions. We employed a sliding window algorithm to identify the boundaries of transcriptionally active regions (step 1 in Figure 1).

**Figure 2:**
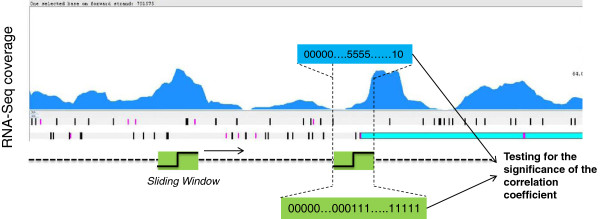
**Identification of putative transcription start points.** A window slides across the nucleotide sequence of the coverage depth file and finds segments of coverage depth highly and statistically correlated with a vector of 100 integers modeling a simple shape of sharp increases in transcription (x = [0_50_,1_50_]). For each highly and statistically correlated segment, we select the center point to indicate a potential transcription start point.

This algorithm uses fixed windows with a length of 100 nt that slides across the nucleotide sequence of the coverage depth file and finds segments of coverage depth highly and statistically correlated with a vector of 100 integers modeling a simple shape of sharp increases in transcription (*x =* [0_50_,1_50_]). In this way, the segments having a positive correlation coefficient (exceeding 0.7) and a significant correlation test p-value (<10^-7^) were selected. The vector of the sliding window of 100 integers is a good trade-off between the accuracy of sharp increases/decreases in transcription and the computational costs of the procedure. P-value 10^-7^ allows reliable identification of sharp increases/decreases in transcription. Figure 2 reports a visual representation of the sliding window process.

We also considered the points with a negative correlation coefficient (< -0.7), for they represent sharp decreases in the transcription. We finally analyzed these points of sharp increases or decreases in transcription to determine transcription start/end points (TSPs and TEPs). After determining the transcription start/end points, we compared the whole coverage depth with the gene annotations in order to estimate the expression level of annotated coding sequences (CDS regions) and intergenic non-coding sequences (IGR regions). The coverage depth was normalized with the RPKM method [1] to allow a comparison in terms of relative expression levels between different genes within the same RNA-seq experiment. Since RPKM values are log-normally distributed, it is convenient to express them as log_2_(RPKM). The RPKM method is able to eliminate the influence of different gene length and sequencing discrepancy when calculating the expression of genes.

### Explanation of operon structures

By linking the transcription start/end points to the operons collected in DOOR, we determined a list of putative Operon Start- and End-Points (OSPs and OEPs). The OSPs were selected using the following criteria: i) a significant change (greater than twofold) of read coverage occurred between the ends of the sliding window ii), the downstream gene was transcribed and the corresponding expression level was higher than a selected minimum expression threshold for CDS regions (*e.g.*, the 10^th^ percentile in the distribution of log_2_(RPKM) for CDS regions), iii) the corresponding gene matched a structural gene of an operon in DOOR, and iv) there was enough space for the 5′UTR. In the same way we selected OEPs (step 2 in Figure 1). Each operon start site was linked to a confirmed operon, therefore we defined a linkage process (step 3 in Figure 1) that adds the next structural genes of an operon until one of the following rules was not verified: first the expression level of the intergenic region was higher than the minimum expression threshold for the IGR regions, second the expression level of the next gene was higher than the minimum expression threshold for the CDS regions, third the intergenic region was not characterized by transcription start/end points. This linkage process allowed determining small sets of operons collected from DOOR and confirmed by experimental data.

**Figure 3:**
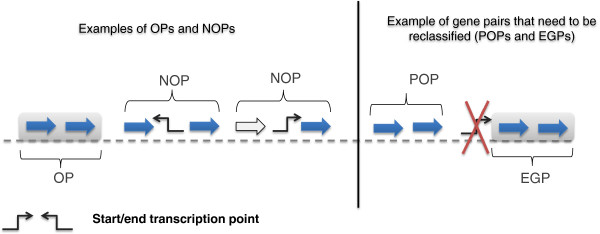
**Examples of OPs, NOPs, EGPs and POPs.** A set of cases of OPs and NOPs. Gray boxes indicate genes in the same operon annotated in DOOR. Blue and white arrows represent genes that are expressed and not expressed for a particular cut-off value, respectively.

In this linkage process it is not necessary to verify the presence of transcription end points after the last added structural gene. These set of confirmed operons were used to select operon pairs (OPs) and non-operon pairs (NOPs). OPs are gene pairs with genes located on the same DNA strand, one next to the other, transcribed together and annotated in DOOR. NOPs are two genes that are adjacent, transcribed in the same direction and with a point of start/end transcription into the corresponding intergenic region. In the list of NOPs we did not verify whether the two genes were transcribed or not. Figure 3 shows the difference between OPs and NOPs.

The lists of OPs and NOPs were used for training and validation. Finally, we identified gene pairs with an operon status to re-define adjacent genes expressed and annotated as NOPs (putative operon pairs - POPs) in DOOR, and adjacent genes expressed, not linked to an identified transcriptional operon start point, and annotated as OPs (expressed gene pairs - EGPs) in DOOR. POPs include also gene pairs representing extensions to known operons; gene pairs formed by the last structural gene of an operon annotated in DOOR and the next gene not associated with an operon. POPs and EGPs, with respect to experimental data, constitute for the DOOR annotations potential false negative and false positive cases. Therefore, we trained and validated three models for operon classifications in order to make reliable re-classifications of gene pairs with an uncertain operon status. In technical terms these cases were treated as unlabeled gene pairs.

### Genomic and transcriptomic features

Several computational methods predict operons based on the properties of adjacent genes, which they try to identity as either OPs or NOPs. Often, the distances between genes and, generally, genomic comparative features have been used for predicting operons. One of the aims of this work was to indicate that transcriptomic features, extracted from RNA-seq based transcriptome profile, can improve the accuracy of prediction models based on standard DNA sequence features. Therefore, we selected two features extracted from the genome sequence, namely the intergenic distance and the codon usage feature as well as two features extracted from RNA-seq data, namely the difference in expression levels of adjacent genes and the expression level within the intergenic region.

#### *Intergenic distance*

The Intergenic distance represents the number of base pairs separating two consecutives genes. A distance-based operon prediction technique was first described by Salgado et al., [5]. Using the genomic sequence of *E. coli* K-12, the authors found that the distribution of the distances between adjacent genes in operons differs from the distribution of distances between adjacent genes at the boundaries of transcriptional units. Therefore, we decided to adopt the distance between two consecutive genes as the first genomic feature for our classification models:

(1) $$\mathrm{igrLength}\left(g_{i},g_{i+1}\right)=g_{i+1}\left(\mathrm{start}\right)+g_{i}\left(\mathrm{end}+1\right)$$

where *g*_*i*_ and *g*_*i+1*_ are two consecutive genes in the genome and the arguments *start* and *end* represent the start and end position of the two adjacent genes in the genome, respectively.

#### *Codon usage features*

It is well known that the genes in the same operon often exhibit similar codon-usage patterns while genes in different operons exhibit different codon bias [20]. Consequently, it may be helpful to consider the codon usage of the genes in order to evaluate whether two consecutive genes constitute an operon pair, [21]. We associated a vector of Relative Synonymous Codon Usage bias (RSCU value) to each gene *g*_*i*_ that specifies a RSCU value for each aminoacid *a*: . Using the RSCU values, we defined the following codon usage feature:

(2) $$\mathrm{RSCU}\left(g_{i},g_{i+1}\right)=\underset{a}{\sum }\mathrm{RSC}U_{a}\left(g_{i}\right)*\mathrm{RSC}U_{a}\left(g_{i+1}\right)\;$$

This measure is symmetric and indicates the consistency and degree to which the bias vectors are characterized by a similar codon bias. RSCU is a simple measure of non-uniform usage of synonymous codons in a coding sequence [22].

#### *Difference in expression levels*

The difference of expression level between two consecutive genes represents the first selected feature extracted from a RNA-seq transcriptome profile.

(3) $$\mathrm{diffExpr}\left(g_{i},g_{i+1}\right)=\mathrm{abs}\left(\mathrm{lo}g_{2}\left(\mathrm{RPKM}\left(g_{i}\right)\right)-\mathrm{lo}g_{2}\left(\mathrm{RPKM}\left(g_{i+1}\right)\right)\right)$$

Genes that are transcribed as a part of an operon should exhibit a similar transcription level, because these genes were in the same transcript. Therefore, two consecutive genes constituting an operon pair should have a difference close to zero. While for a non-operon pair this feature should exhibit a high value of mean and variance. Our results confirmed that this feature is a good discriminator.

#### *Expression level of intergenic regions*

The expression level of the intergenic regions represents the second transcriptomic feature that we used for our prediction models. If the intergenic region between two consecutive genes is highly expressed and these genes are transcribed in the same direction with a similar expression value, than they can be part of an operon structure. We compared the RNA-seq based transcriptome profile with the available genome annotation to identify the expression level of intergenic regions.

(4) $$\mathrm{igrExpr}\left(g_{i},g_{i+1}\right)=\mathrm{lo}g_{2}\left(\mathrm{RPKM}(\mathrm{igr}\left(g_{i},g_{i+1}\right)\right)$$

The *igrExpr()* function takes as input the intergenic region between *g*_*i*_ and *g*_*i+1*_. We allowed the expression levels derived from intergenic sequences of adjacent genes in the same operon to be higher than the expression levels of intergenic sequences between genes that are not in the same operon. Indeed, the intergenic expression level of con-firmed OPs resulted to be almost always greater than the intergenic expression level of NOPs. More details are given as Additional file 2.

### Models validation

Using the selected features, we defined a dataset of confirmed operon and non-operon pairs and evaluated the performance of three classification models: RFs, NNs and SVMs. We compared these three models, because they are the most recently and performing machine-learning methods used for operon prediction [8,23-25].

All prediction models were trained specifically and separately for each organism in each RNA-seq transcriptome profile. We randomly selected and held out the 30% of the dataset patterns as a test set and used the rest (70%) for a 5-fold cross validation procedure. For each task, we executed 10 runs of a 5-fold validation. We used the R package *rminer*[26] to accomplish these tasks. In *rminer*, the NN and SVM hyper-parameters (*e.g*. H) are optimized using a grid search. To avoid overfitting, the training data is further divided into training and validation sets (holdout) or, alternatively, an internal k-fold is used.

We did not run cross validation in RFs, as the test set error is internally estimated. However, in order to compare the different classifiers, we run our RF-based model for ten times on the training set and calculated every time the evaluation metrics. Finally, the three models were compared on the test set.

#### *Evaluation metrics*

The following metrics were used to compare the performance of the three different classifiers:

• True Positive Rate (TPR): TP/(TP + FN).

• Positive predictive value or Precision (PPV): TP/(TP + FP).

• False Positive Rate or Recall (FPR): FP/(FP + FN).

• Error rate (ER): (FP + FN)/(TP + FN + TN + FP).

• Accuracy (ACC): (TP + TN)/(TP + TN + FP + FN).

Where: TP (True Positives) = Number of OPs accurately classified as operon pairs by the model; FN (False Negatives) = Number of OPs falsely classified as non-operon pairs by the model; FP (False Positives) = Number of NOPs falsely classified as operon pairs; TN (True Negatives) = Number of NOPs accurately classified as non-operon pairs. Recall quantifies the sensitivity of the model, *i.e.* how many OPs could be predicted as operon pairs by the model, and precision quantifies the specificity of the model, *i.e.* how many of the operon pairs predicted from the training set (OPs and NOPs) were in fact OPs. Then, the error rate is the percentage of errors made over the whole set of instances (records) used for testing. Finally, the accuracy is the percentage of well-classified data in the testing set.

#### *K-fold cross validation and ROC curves*

In the 5-fold cross-validation, the data is randomly split into five subsets (called 5-folds) of the same size. The first four folds are used for training, while the remaining fold is used for testing the classifier. In our analysis, the 5-fold cross-validation was performed 10 times (10 × 5) and the true classes of the gene pairs in each of the 50 test subsets were then used to generate receiver operating characteristics (ROC) graphs.

### Promoters and terminators

The transcription of a unit encoding a single gene or an operon is controlled by a promoter and a terminator. Therefore, in order to increase the reliability of gene pairs classified as an operon pair, we verified the absence of any promoter or terminator in the corresponding intergenic regions. The promoters and terminators were predicted across the genome, using PromPredict [27], Pepper (Prediction of Prokaryote Promoter Elements and Regulons) [28], and TransTermHP [29], in order to add confidence to the identified novel operon pairs. PromPredict can identify putative promoters using the whole-genome percentage GC of selected bacterial genomes. The Pepper Toolbox provides an improved promoter prediction for prokaryotes based on curated PWM and HMMs models. The training of HMMs is based on DBTBS, RegulonDB and MolGen. Finally, TransTermHP finds rho-independent transcription terminators in bacterial genomes.

## Results and discussion

We have developed a novel prediction method that successfully infers condition-dependent operons. Our approach supports the idea that RNA-seq transcriptome profiles are not sufficient to detect operons, even when using accurate TAR detection algorithm. Although RNA-Seq experiments are regularly more accurate than other similar high-throughput technologies, they still exhibit a high error rate. These errors can have a large impact on the downstream bioinformatics analysis and lead to wrong conclusions regarding the set of transcribed mRNAs (or identified operons). Therefore, only by integrating multiple and complementary sources of information (static data from DOOR and dynamic data from RNA-seq), it is possible to improve the current operon prediction accuracy and provide condition-dependent operon predictions.

A similar operon prediction method has been recently reported [35] and included in a tool for analyzing bacterial RNA-seq data, called Rockhopper (http://cs.wellesley.edu/~btjaden/Rockhopper/). There, the authors have investigated the extent to which operon structures can be predicted using the intergenic distance and the correlation of gene expression across RNA-seq experiments. While this strategy requires replicated transcriptome profiles, our approach aims at training operon classifiers using both DNA-sequence based information as well as expression-based features that can be also extracted from a single transcriptome profile.

In order to evaluate the performance of our method, we tested it on different RNA-seq datasets [14,15,35] of four different species of bacteria (see Tables 1 and 2). The compiled RNA-seq expression profiles were based on total RNA samples isolated from different laboratory culturing conditions, and the mapped RNA-seq reads covered both coding and non-coding sequence regions. In the first study, the authors used a standard RNA-seq method construct a single nucleotide resolution of the *H*. *somni* transcriptome (strain 2336, here indicated as HS). In the second study, the authors applied a strand-specific RNA-seq protocol to characterize the transcriptome of the periodontal pathogen *P. gingivalis* (strain W83) under three different experimental growth conditions, here called PG1, PG2 and PG3. The third study was considered in order to benchmark our method using RNA-seq datasets compiled from well-known bacteria, such as *E. coli* (strain K-12 substrain MG1655) and *S. enterica* (strain LT2). All the predicted condition-dependent operons were compared with the prediction obtained by Rockhopper [35].

**Table 1:** General information of microbial genomes used for testing

**General Information**	***H. somni***	***P. gingivalis***	***E. coli***	***S. enterica***
Annotated genes	2,065	2,053	4,669	4,606
Annotated operons (DOOR)	464	445	865	879
Genes in operons	~ 70%	~ 68%	~ 60%	~ 60%

For each case study, the number of known or predicted genes, the number of operons collected by DOOR and the percentage of gene pairs predicted as OPs are reported.

**Table 2:** RNA-seq datasets considered in this study

**Genome**	**Conditions**	**Description (growth condition)**	**Code**	**Accession**
*H. somni*	1	A single nucleotide resolution transcriptome profile	HS	GSE29578
*P. gingivalis*	3	MIN - Defined minimal medium	PG1	GSE30452
		TSB - Trypticase soy broth	PG2	
		BAPH - Sheep blood agar	PG3	
*E. coli*	2	WT in LB + αMG	EC1	GSM1104387-89
		WT in LB –αMG	EC2	GSM1104390-92
*S. enterica*	2	WT (15 minutes after αMG stress)	SE1	GSM1104438
		WT (30 minutes after αMG stress)	SE2	GSM1104439

There are two advantages with the proposed integrative approach. The first one concerns with the classical approach of identifying operons in prokaryotic genomes. In the DOOR database, operon maps predicted using features extracted from genome DNA sequence (e.g., intergenic distance) of well-characterized genomes, such as *E. coli* and *B. subtilis* are collected. It has been proposed that “distance models” for predicting operons can be transferred from one species to other unrelated species, but this *ad hoc* approach has only been validated for *E. coli* and *B. subtilis*[6]. A subsequent study indicated that, in general, intergenic distances within conserved operons vary across species [30]. Thus the distance model of *E. coli* may not always be effective in predicting operons in all other prokaryotic genomes. On the contrary, the distance models should be trained on the organism for which the predictions of condition-dependent operon maps are performed. Hence, the set of confirmed operons identified in our approach from the transcriptome analysis is necessary to model the specific properties (*e.g.*, intergenic distance) of the investigated genome. These DNA-dependent sequence properties can be used to train/validate new classification models that, in their turn, are required to identify the remaining operons with similar characteristics.

The second advantage of our method comes from the use of expression-based features extracted from RNA-seq transcriptome profile. As a matter of fact, RNA-Seq can be readily used to identify transcriptionally active regions, which in turn can be used to identify operons/TUs through a mapping process. Methods for the identification of transcripts from RNA-seq mapped reads are based on the assumption that the reads of RNA-Seq are uniformly distributed along the transcripts [31,32]. However, the reads are usually non-uniformly distributed along the transcripts, which can greatly reduce the accuracy of these methods based on the uniform assumption [33]. Furthermore, commonly used RNA-seq strategies can result in transcript-length bias because of the multiple fragmentation and RNA or cDNA size-selection steps they use [34]. This bias may result in difficult transcriptome mapping based approaches, above all when we desire to map longer features, such as the transcripts of a given operon. Consequently, the identification of operons using only accurate TAR detection algorithms is not recommended. While, the use of simple RNA-seq based transcriptome features (see Methods) compiled from a set of confirmed operons, allow to substantially improve the accuracy of conventional operon prediction methods.

### Empirical evaluation

All the prediction models were trained specifically and separately for each organism in each RNA-seq transcriptome profile. Therefore, given the genome sequence and a RNA-seq profile, a set of confirmed OPs, NOPs and POPs was defined (see “Explanation of Operon Structures” in Methods).

Subsequently, the set of OPs and NOPs were used to build the dataset for the training and validation steps (see “Genomic and transcriptomic features” in Methods). We used this dataset to separately train and validate the RF-, NN- and SVM-based classifiers. Table 3 shows the number of confirmed and annotated OPs and NOPs that have been used to generate the training and test datasets. From each list of OPs and NOPs, we randomly selected the 30% as the test set and used the rest (70%) for a 5-fold cross validation. During the cross-validation step we computed several evaluation metrics to assess the performance of our classifiers. For the RF-based models we simply run the training process ten times and calculated every time the evaluation metrics (for more details, please see “Models validation” in Methods). A comparison of all the accuracy values for each transcriptome RNA-seq profile is reported in Table 4. For each classifier we reported the mean and the standard deviation of the accuracy values computed over bootstrap samples.

**Table 3:** Adjacent gene pairs with a confirmed operon status

**Confirmed OPs and NOPs**	***H. somni***	***P. gingivalis***			***E. coli***		***S. enterica***	
***Conditions***	***HS***	***PG1***	***PG2***	***PG3***	***EC1***	***EC2***	***SE1***	***SE2***
OPs	101	121	124	124	214	232	232	276
NOPs	168	66	53	45	94	100	191	200
#Operons	78	73	69	68	143	137	146	158

For each transcriptome RNA-seq profile the number of confirmed and annotated OPs and NOPs are indicated. These confirmed OPs and NOPs are necessary to build the training/validation and test sets.

**Table 4:** Accuracy values from the 5-fold cross validation process, using all the features

**Genome**	**Condition**	**Dataset**	**NNs**	**RFs**	**SVMs**
*H. somni*	HS	Training set	0.95 (0.01)	0.97 (0.008)	0.97 (0.006)
		Test set	0.98	0.99	0.96
*P. gingivalis*	PG1	Training set	0.98 (0.002)	0.97 (0.004)	0.96 (0.004)
		Test set	0.99	0.98	0.95
	PG2	Training set	0.98 (0.003)	0.99 (0.004)	0.97 (0.003)
		Test set	0.98	0.98	0.98
	PG3	Training set	0.98 (0.001)	0.97 (0.002)	0.97 (0.002)
		Test set	0.98	0.98	0.90
*E. coli*	EC1	Training set	0.98 (0.009)	0.98 (0.003)	0.92 (0.008)
		Test set	0.98	0.99	0.95
	EC2	Training set	0.98 (0.003)	0.98 (0.006)	0.94 (0.009)
		Test set	0.99	0.99	0.99
*S. enterica*	SE1	Training set	0.95 (0.01)	0.97 (0.001)	0.91 (0.006)
		Test set	0.97	0.98	0.92
	SE2	Training set	0.94 (0.02)	0.98 (0.004)	0.90 (0.01)
		Test set	0.98	0.98	0.93

The accuracy values obtained with training (5-cross validation) and testing datasets are shown. The values are aggregates from all five folds.

Results displayed good performance for all the models, with accuracy values ranging in 90-99% on the training sets. We also noted that, with strand-specific RNA-seq data, the trained models could reach 99% accuracy.

Other two important metrics are the precision and the recall. The precision quantifies the specificity of the model, which is how many operon pairs predicted from the training set are annotated in DOOR and confirmed by RNA-seq data. On the other hand, the recall quantifies the sensitivity of the model, which is how many annotated and confirmed OPs can be predicted as operon pairs by the models. In our tests, we obtained an average precision ranging in [0.97-0.98] for NN-based classifiers, in [0.95-0.99] for RF-based classifiers and in [0.95-0.97] for SVM-based classifiers; for what concerns the recall metric we achieved values ranging in [0.97-1] for NNs, in [0.92-0.99] for RFs and in [0.94-1] for SVMs.

**Figure 4:**
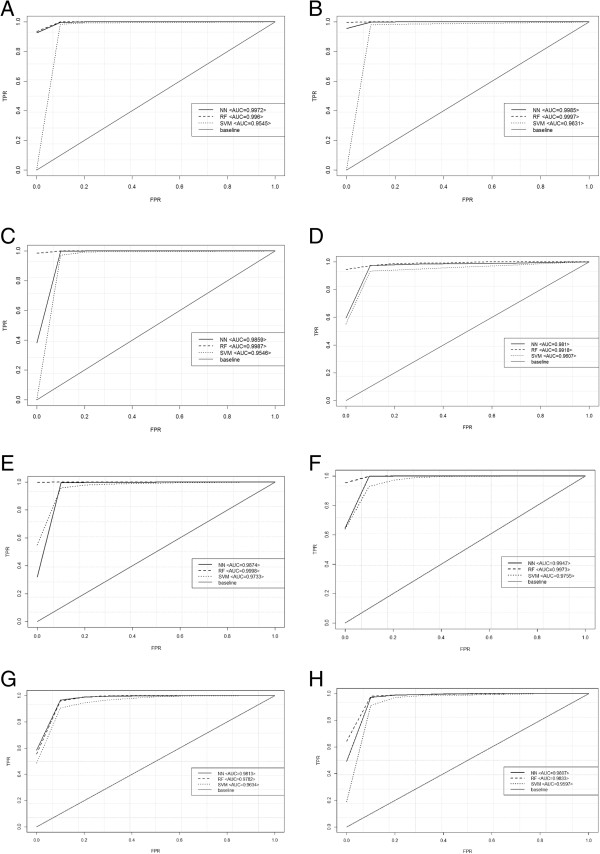
**ROC Curves.** Comparison of different classifiers in each RNA-seq transcriptome profile by ROC curves. We used the ROC Curve analysis to compare the accuracy of the three classification models trained, every time, on a set of different, confirmed OPs/NOPs in PG1 **(A)**, in PG2 **(B)**, in PG3 **(C)** in HS2336 **(D)**, in EC1 **(E)**, in EC2 **(F)**, in SE1 **(G)** and in SE2 **(H)**.

Figure 4 show the ROC curves that we generated to evaluate the overall accuracy of the three classification methods. The ROC curves display the full picture of the trade-off between sensitivity (TPR) and “1-specificity” (FPR) across a series of cut-off points. All the prediction models showed very good results. Furthermore, we noted that also with the RNA-seq transcriptome profile obtained with strandness reads, the accuracy was greater than 95%, suggesting that our method is valuable for strand- and not strand-specific RNA-seq experiments as well as robust enough to yield a reasonable predictive performance about new potential operon pairs. The graphs in Figure 4 include also AUC values.

### Sensitivity Analysis to measure input importance

**Figure 5:**
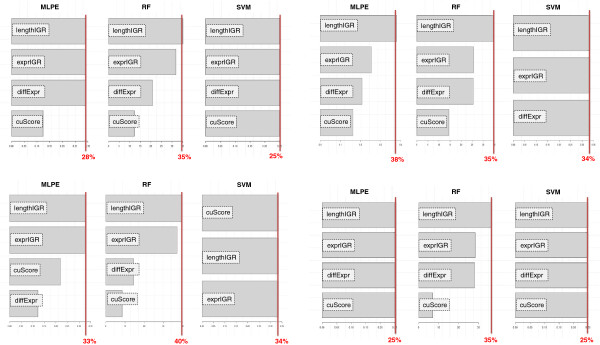
**1-D input importance computed for some models.** Bar plots with the 1-D input importance computed for some of the RNA-seq based transcriptome profiles. We measured the importance of each feature in each supervised model that we trained/validated, using a sensitivity analysis (SA), which is a simple method that measures the effects on the output of a given model when the inputs are varied through their range of values. The red vertical bar indicates the highest sensitive score reached by features.

Sensitivity Analysis (SA) is a simple method to measure the effects on the output of a given model when the inputs are varied through their range of values [36]. This method allows a ranking of the inputs that is based on the amount of output changes that are produced due to small variation in a given input [37]. In our analyses, we used a computationally efficient one-dimensional (1-D) SA method implemented into *rminer*, where only one input was changed at the time, being the remaining ones hold at their average values. In these experiments, the input importance was measured as the variance of the responses, indicating that the transcriptomic features extracted from RNA-seq data can be used to improve the prediction of operons in prokaryotes (Figure 5). We observed that effectively the two selected transcriptomic features, *exprIGR* and *diffExpr*, are important features to classify OPs and NOPs. Besides, we also noted that the *cuScore* feature is less useful in classification of OPs and NOPs.

### Performance of different groups of features

We evaluated the classification performance of different subsets of features in order to compare the classifiers based on genomic features with those based on transcriptomic features. When comparing the accuracy values, we found that the combination of all the selected features resulted in the best classification performance in all the datasets (Table 5). If only the expression data was used for classification, the average accuracy ranged in [0.87-0.92]. On the other hand, if only genomic properties were considered, the average accuracy value ranged in [0.86-0.95]. Therefore, the models trained with standard DNA sequence features perform marginally better than the models trained with expression features alone. However, it was clear that when using genomic with expression features together we could achieve higher levels of accuracy (ranging in [0.97-0.99]).

**Table 5:** The contribution of transcriptomic features in improving the classification accuracy

**Dataset**	**All**			**Genomic**			**Transcriptomic**			**IGR, CuScore**			**IGR, CuScore**		
				**Features**			**Features**			**and IGR-Expr**			**and Diff-Expr**		
	**NN**	**RF**	**SVM**	**NN**	**RF**	**SVM**	**NN**	**RF**	**SVM**	**NN**	**RF**	**SVM**	**NN**	**RF**	**SVM**
PG1	0.98	0.97	0.97	0.9	0.89	0.9	0.89	0.87	0.88	0.96	0.96	0.96	0.91	0.94	0.87
PG2	0.99	0.99	0.97	0.95	0.95	0.92	0.9	0.9	0.91	0.97	0.97	0.96	0.97	0.96	0.97
PG3	0.98	0.97	0.97	0.95	0.94	0.9	0.92	0.89	0.91	0.96	0.95	0.95	0.96	0.95	0.96
HS2336	0.95	0.97	0.97	0.86	0.86	0.86	0.89	0.88	0.89	0.94	0.96	0.95	0.88	0.89	0.88

Four subsets of features have been tested and compared by the corresponding accuracy values. The first column reports the accuracy results with all the features. The next two columns show the accuracy values achieved, respectively, with genomic and transcriptomic features. Finally, the last two columns display the improvement, in classification accuracy, obtained combining one transcriptomic feature to the two genomic features.

Some discrepancies in the classification results occurred between strand- and not strand specific dataset. This is likely due to the nature of RNA-seq data that we considered in our study. Since strand-specific RNA-seq protocols have the advantage of reducing the noise compared to the not strand-specific RNA-Seq protocol, we expect to have higher accuracy values for operon prediction models trained on strand-specific data. For instance, when using not strand-specific RNA-seq data, we observed that the operon prediction models based on genomic features are characterized by low accuracy values (86%). Nevertheless, the operon models based on a combination of genomic and transcriptomic features shown higher levels of accuracy that are similar to those obtained using strand-specific RNA-Seq data. Finally, we can state that the expression features extracted from RNA-seq data are an important factor for determining whether two adjacent genes represent an operon pair or not. Clearly, the discrimination power of these features depends on the quality of RNA-seq data in terms of sequencing depth, strand specificity, coverage uniformity, and read distribution over the genome structure.

### Performance on different datasets

In this section we show how our method performs when a classifier is trained on one dataset and tested on different data sets of different conditions/organisms. We defined a simple validation test aiming to demonstrate that the classification accuracies computed on different datasets is significantly lower than those obtained with the original datasets. Each trained classifier was tested on 50 bootstrap samples (70%) compiled from the original datasets and from a different dataset in order to evaluate the corresponding accuracy values through a t-test. Table 6 reports for each test the mean accuracy of the RF-based classifiers tested on different datasets and the corresponding p-values. For this test we considered only the RNA-seq data sets of *E. coli* and *S. enterica*. As expected, the differences are always statistically significant and the accuracy values are lower than those obtained with the original dataset. Moreover, these differences are larger even when a dataset defined on the same genome, but in a different condition, was considered. This supports the idea using computational approaches that are able to provide condition-dependent operon predictions.

**Table 6:** Accuracy values obtained with RF-classifiers tested on different datasets

**Genome**	**Condition**	**Original dataset**	**Different condition**	**Different genomes/conditions**	
		*EC1*	*EC2*	*SE1*	*S2*
*E. coli*	*EC1*	0.98	0.84 (0.001)	0.83 (0.004)	0.83 (0.01)
		*EC2*	*EC2*	*SE1*	*SE2*
	*EC2*	0.98	0.86 (0.001)	0.83 (0.004)	0.81 (0.01)
		*SE1*	*SE1*	*EC1*	*EC2*
*S. enterica*	*SE1*	0.97	0.93 (0.001)	0.92 (0.01)	0.91 (0.001)
		*SE2*	*SE2*	*EC1*	*EC2*
	*SE2*	0.98	0.87 (0.001)	0.85 (0.02)	0.86 (0.004)

The accuracy values obtained performing a classifier trained on one dataset and tested on different data sets of different conditions and different organisms. Each test was conducted running the RF-based classifier on 50 bootstrap samples compiled from a different dataset and using the corresponding accuracy values to execute a t-test. All the p-values were less than 0.001.

### Combining operon prediction results

A simple majority voting schema (SMVS) was adopted to combine the classification predictions and to improve the accuracy. The voting system tags a gene pair as an OP when at least two classifiers have predicted that gene pair as an OP.

$$\begin{array}{l} \mathrm{RF}\left(x\right)\;\mathrm{NN}\left(x\right)\;\mathrm{SVM}\left(x\right)\\ \begin{array}{ccc} 0 & 1 & 1\\ 1 & 1 & 1\\ 1 & 0 & 1 \end{array}\begin{array}{c} \to \\ \to \\ \to \end{array}\begin{array}{c} x\in \mathrm{class}\;\mathrm{OP}\\ x\in \mathrm{class}\;\mathrm{OP}\\ x\in \mathrm{class}\;\mathrm{OP} \end{array}\\ \begin{array}{ccc} 1 & 1 & 1\begin{array}{cc} \to & x\in \mathrm{class}\;\mathrm{OP} \end{array} \end{array}\\ \begin{array}{cc} \mathrm{Otherwise} & x\in \mathrm{class}\;\mathrm{NOP} \end{array} \end{array}$$

Where *x* represents two adjacent genes and *RF(x) = 1*, for instance, indicates that the RF-based model classified x as an OP. The SMVS gives the prediction accuracy between 98% and 100%. Furthermore, we used this voting system to predict the class of gene pairs with an operon status to redefine (POPs and EGPs), and also to build the condition-dependent operon map in each RNA-seq based transcriptome profile. Condition-dependent operon maps were defined through a linkage process that finds adjacent genes predicted as OPs and groups them into operons. Table 7 reports a summary of the condition-dependent operon pairs found by our approach in each RNA-seq transcriptome profile. The Table 8 indicates the number of condition-dependent operon pairs found using the proposed approach compared to the number of operon pairs found by Rockhopper in each RNA-seq experimental condition.

**Table 7:** Summary of predicted condition-dependent operons

**Genome**	**Dataset**	**Confirmed**	**Tot. Confirmed**	**Putative**	**Total number of identified operons**
*P. gingivalis*	PG1	438 (49%)	510 (57%)	30	468
	PG2	473 (53%)		26	499
	PG3	502 (56%)		35	537
*H. somni*	HS	416 (43%)	416 (43%)	27	443
*E. coli*	EC1	1390 (79%)	1412 (80%)	47	1437
	EC2	1284 (73%)		37	1321
*S. enterica*	SE1	1324 (73%)	1385 (76%)	126	1450
	SE2	1305 (72%)		120	1425

The number of operon pairs that our method found in each transcriptome profile are reported. The column “Confirmed” indicates the number of operon pairs that we found in each condition. While, the column “Tot. Confirmed” denotes the total number of confirmed operon pairs found considering all the conditions. The rest of the columns contain the number of new potential operon found and the total number of operons provided in each experimental condition.

**Table 8:** Summary of comparisons to Rockhopper’s operon identifications

**Genome**	**Dataset**	**Replicates**	**Confirmed**		**Tot. Confirmed**	
			**R**	**P**	**R**	**P**
*P. gingivalis*	PG1	1	85%	49%	94%	57%
	PG2	1	85%	53%		
	PG3	1	85%	56%		
*H. somni*	HS	1	NA	43%	NA	43%
*E. coli*	EC1	3	79%	79%	90%	80%
	EC2	3	79%	73%		
*S. enterica*	SE1	1	83%	73%	91%	76%
	SE2	1	83%	72%		

The table summarizes the comparisons concerning the confirmed condition-dependent operon predictions obtained using the proposed approach (P) and Rockhopper (R). We run ROCKHOPPER on each condition separately and then we run Rockhopper on each set of conditions for each bacterial organism. The available format of the raw data for *H. somni* is not supported in Rockhopper.

### Comparison with Rockhopper

We explored the possibility that Rockhopper could be used for condition-dependent operon prediction, similar to our approach. To this end, we used three different datasets described earlier, namely the datasets for *E. coli* and *S. enterica* with two and for *P. gingivalis* with three different experimental conditions. We excluded *H. somni* dataset from this comparison, as the RNA-seq data were available only in bed-file format, which is compatible with our method but not with Rockhopper. We observed that, in each comparison/condition, the number of operon pairs confirmed by Rockhopper is higher than the number of operon pairs confirmed by our computational approach. In part this result was expected because the proposed technique cannot be applied to genes not significantly expressed under the studied condition, therefore some operon pairs are missing. However, Rockhopper returned identical predictions between the different experimental conditions, confirming that the current version of Rockhopper is not optimized for condition specific operon prediction.

### Identification of intergenic/control signals

**Figure 6:**
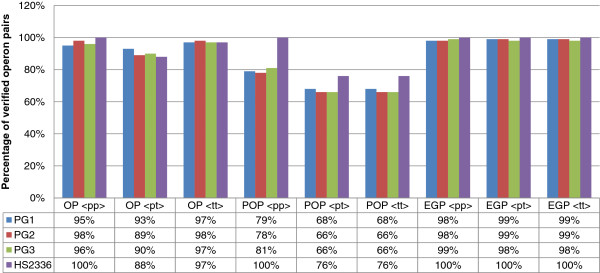
**OP control signals.** Bar plots of the percentages of control signals identified in gene pairs that have been predicted as OPs by our classification system in *H. somni* and *P. gingivalis*. The percentages are reported for each used prediction program: PromPredict < pp>, Pepper Toolbox < pt > and TransTerm < tt > .

Figure 6 shows the percentage of gene pairs classified as OPs in which we computationally verified the absence of predicted control signals (promoter and terminator) in the corresponding intergenic region. The percentage of operon pairs without promoters/terminators was very high in all the datasets and at least 60% of the putative operon pairs, predicted as OPs, do not have a promoter or terminator in the corresponding intergenic regions. Several authors have suggested that the identification of signals occurring on the boundaries of an operon, such as the promoter and the terminator, does not improve the accuracy of the operon predictions [38]. Hence, we did not verify the presence of control signals in the flanking regions of the operon pairs, also because they could be part of a larger unit.

### Examples of predicted operons supported by evidence

We identified new operons in *H. somni* and *P. gingivalis* that were incorrectly reported in DOOR but were experimentally defined also in other transcriptome studies. For instance, a new operon consisting of three genes: HSM1354, HSM1355 and HSM1356, annotated as ribosomal protein L20, ribosomal protein L35, and translation initiation factor IF-3 respectively [15], were found in *H. somni*. The orthologs of these genes are well known to form a functional operon of ribosomal proteins (IF3-L35-L20) in *Escherichia coli*[39]. Similarly, we observed the three genes (PG0375, PG0376 and PG0377) operon annotated as the ribosomal protein L13 (*rplM*), the ribosomal protein S9 (*rpsI*), and the ribosomal protein S2 (*rpsB*) in *P. gingivalis.* Our method reported that PG0375 and PG0376 constitute an operon, while the gene PG0377 forms an operon with the next gene annotated as an elongation factor Ts (PG0378). Bendiak *et al.* (1981) [40] found that *rpsB* and *tsf in Escherichia coli* can be transcribed in one transcriptional unit, with *rpsB* being promoter-proximal. The operon pair *rpsB-tsf* was not identified by Rockhopper. Besides, Isono *et al.*[41] experimentally confirmed that *rplM* and *rpsI* could be co-transcribed in *Escherichia coli*.

Moreover, when testing our pipeline on datasets used in Rockhopper publication we found that several predicted operons, not present in DOOR, were also supported by literature. Examples of such operons in *E. coli* are pairs B0098-B0099 and B1716-B1717. Genes B0098 and B0099 have been found to be in same operon and co-translated [42,43]. There is similar evidence for genes B1716 and B1717 [44]. Similarly we find examples of predicted operons in *S. enterica* data that are not included in DOOR but are supported by evidence in literature such as *malK-lamB* pair [45] and *aspA-dcuA* pair [46].

## Conclusions

We provide a new computational strategy to reveal gene pairs that are co-transcribed in some experimental conditions but not in others, even when just one replicate is available. Our approach should be considered as complementary to other strategies focusing on the definition of single, non condition dependent, ‘optimal’ operon maps. Moreover, the proposed method proves that integrating several sources of information, including DNA sequence properties and features from transcriptionally active regions, NN-, RF- and SVM-based classification algorithms can accurately classify > 98% of the gene pairs. Our results indicate that the combination of DNA sequence data and expression data results in more accurate predictions than either alone. This proves that the transcriptomics data can improve the accuracy of operon prediction methods. Finally, the output of the three classifiers summarized by a voting scheme based system is effective in building accurate condition-dependent operon maps as well as identifying new potential operons and extensions to pre-existing operons.

## Abbreviations

CDS: Coding Sequence region; IGR: Intergenic region; TSP/TP point: Transcription Start Point/Transcription End Point; OSP: Putative operon start point; OEP: Putative operon end point; OP: Operon pair; NOP: Non-operon pair; POP: Putative operon pair; EGP: Expressed gene pair; RSCU: Relative synonymous codon usage bias; NN: Neural network; RF: Random forest; SVM: Support vector machine.

## Competing interests

The authors declare that they have no competing interests.

## Authors’ contributions

VF conceived the project, implemented and tested the method, analysed the data and drafted the manuscript. OS and PA participated in the data analysis and interpretation of the results. RT and DG supervised the project and analysed the results. All authors have read and approved the final manuscript.

## Supplementary Material

Additional file 1 Zip-file containing the R-based pipeline that implements the proposed method and some example scripts that show how to use it. Click here for file

Additional file 2 Supplementary information about the methods, the implementation and the results. Click here for file
