# Supplementary material for: Transcriptome dynamics-based operon prediction in prokaryotes
Source: BMC Bioinformatics. 2014 May 16;15:145. doi: 10.1186/1471-2105-15-145 (PMC4235196; doi:10.1186/1471-2105-15-145)
Supplement: Additional file 2 — Supplementary information about the methods, the implementation and the results. [file 1471-2105-15-145-S2.docx]

Index

[1 - Information about the RNA-seq data 2](#_Toc361472285)

[2 - Further details about the “*igrExpr*” feature 2](#_Toc361472286)

[3 - Further details about the features used for the operon classification models 3](#_Toc361472287)

[4 - The RPKM function 4](#_Toc361472288)

[5 - Details about the ROC curves and the input importance bar plots 5](#_Toc361472289)

# 1 - Information about the RNA-seq data

| Accession | GSE30452 | GSE29578 |
| --- | --- | --- |
| Author | Kumar *et al.*, 2012 | Hövik *et al.*, 2012 |
| Organism | *Porphyromonas gingivalis* | *Haemophilus somni* |
| Conditions | 3 | 1 |
| Platform | Illumina Genome Analyzer II | Illumina Genome Analyzer II |
| Library | Strand-specific cDNA | Illumina protocol |
| Sample | PG1 (MIN), PG2 (TSB), PG3 (BAPH) | HS2336 |

This table reports the target organism, number of experimental conditions, the platforms that were used for cluster generation and sequencing, the RNA-seq library preparation protocol and the name of the samples that we will use in the manuscript. P. *gingivalis* W83 was grown in three different laboratory culturing media: a chemically defined minimal medium (MIN), Trypticase soy broth (TSB), and sheep blood agar (BAPHK).

# 2 - Further details about the “*igrExpr*” feature

The intergenic expression level for two adjacent and overlapping genes corresponds to the RPKM expression value computed for the overlapping region. Since the RPKM method penalizes the expression level computed for short sequences, we defined the following function to compile the *igrExpr* feature for two adjacent and overlapping genes:

$$igrExpr\left( g_{i}{,g}_{i+1} \right)=minExpr\left( g_{i}{,g}_{i+1} \right)+w({g_{i},g}_{i+1})$$

where

$$minExpr\left( g_{i}{,g}_{i+1} \right)=min \left( {log}_{2}\left( RPKM(g_{i} \right),{log}_{2}\left( RPKM(g_{i+1} \right) \right)$$

$$w\left( {g_{i},g}_{i+1} \right)=diffExpr\left( g_{i},g_{i+1} \right)*\frac{overlapping\_base\_pairs}{min(length\left( g_{i} \right), length(g_{i+1}))}$$

The $igrExpr$ function uses an increment of the expression level resulting from the overlapping section (indicated as *w*), and adds this increment to the expression level of the gene with the minimum expression value (indicated as minExpr). In this way, the larger is the difference in gene level expression and/or the overlapping section, the greater is the increment value *w(g_i_,g_i+1_)*, and this influences the expression value computed for the overlapping section. Our results evidenced that this feature is a good discriminator between OPs and NOPs (Fig. 1-4).

# 3 - Further details about the features used for the operon classification models

In all the transcriptome profiles, the confirmed OPs have shorter intergenic distance and higher similarity of codon usage bias. In addition, the expression differences between adjacent genes composing operons are close to zero. On the other hand, NOPs have the expression differences that have higher mean values. Furthermore, genes within operons are likely to have a higher expression level in their intergenic regions compared to gene pairs that are labeled as NOP. The presence of particular transcription regulatory elements in the intergenic regions, such as small non-coding RNAs, leads to a minimum level of transcription.


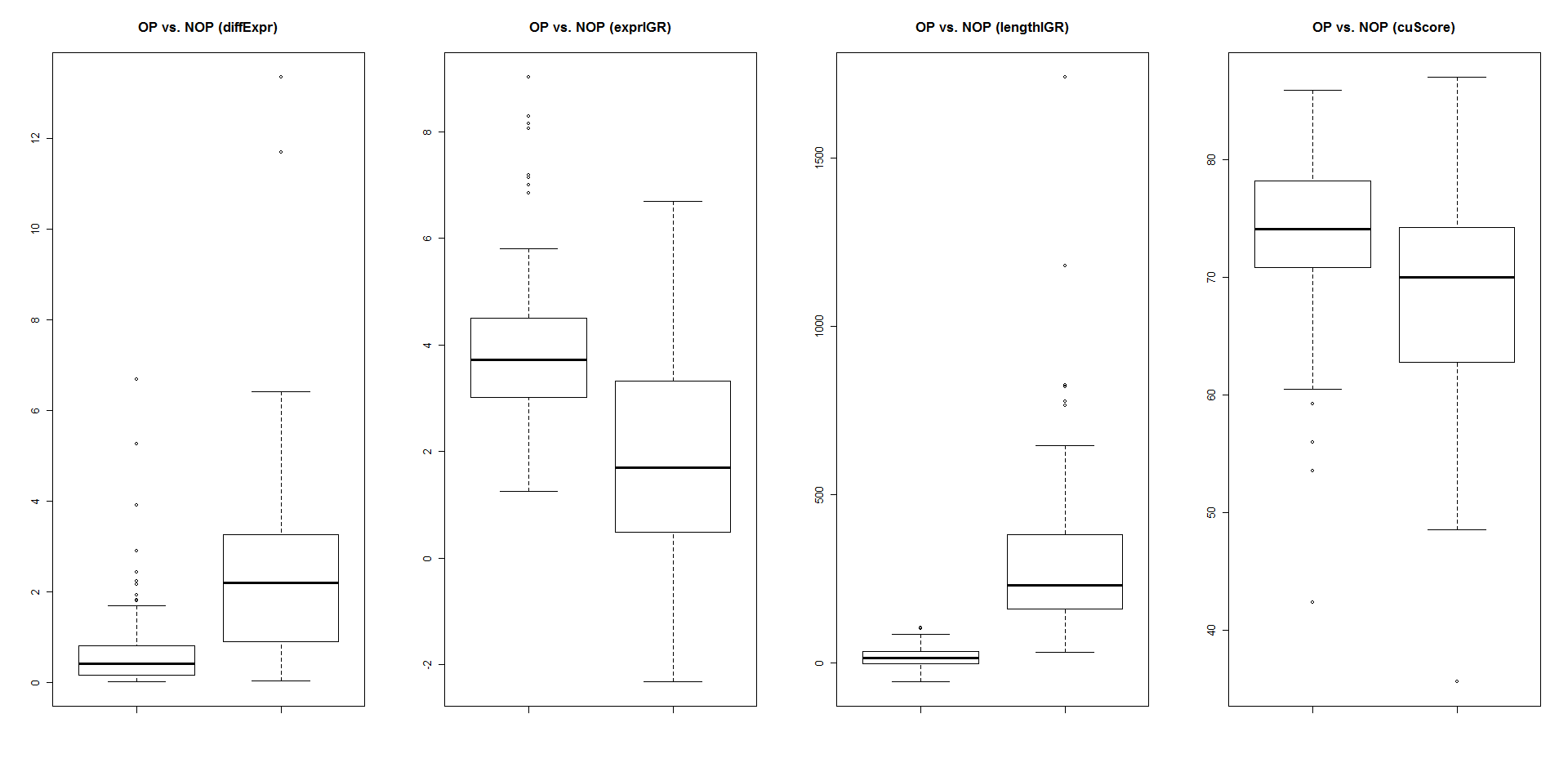


Figure 1 – Box plots showing the feature value distributions for OPs and NOPs in PG1.


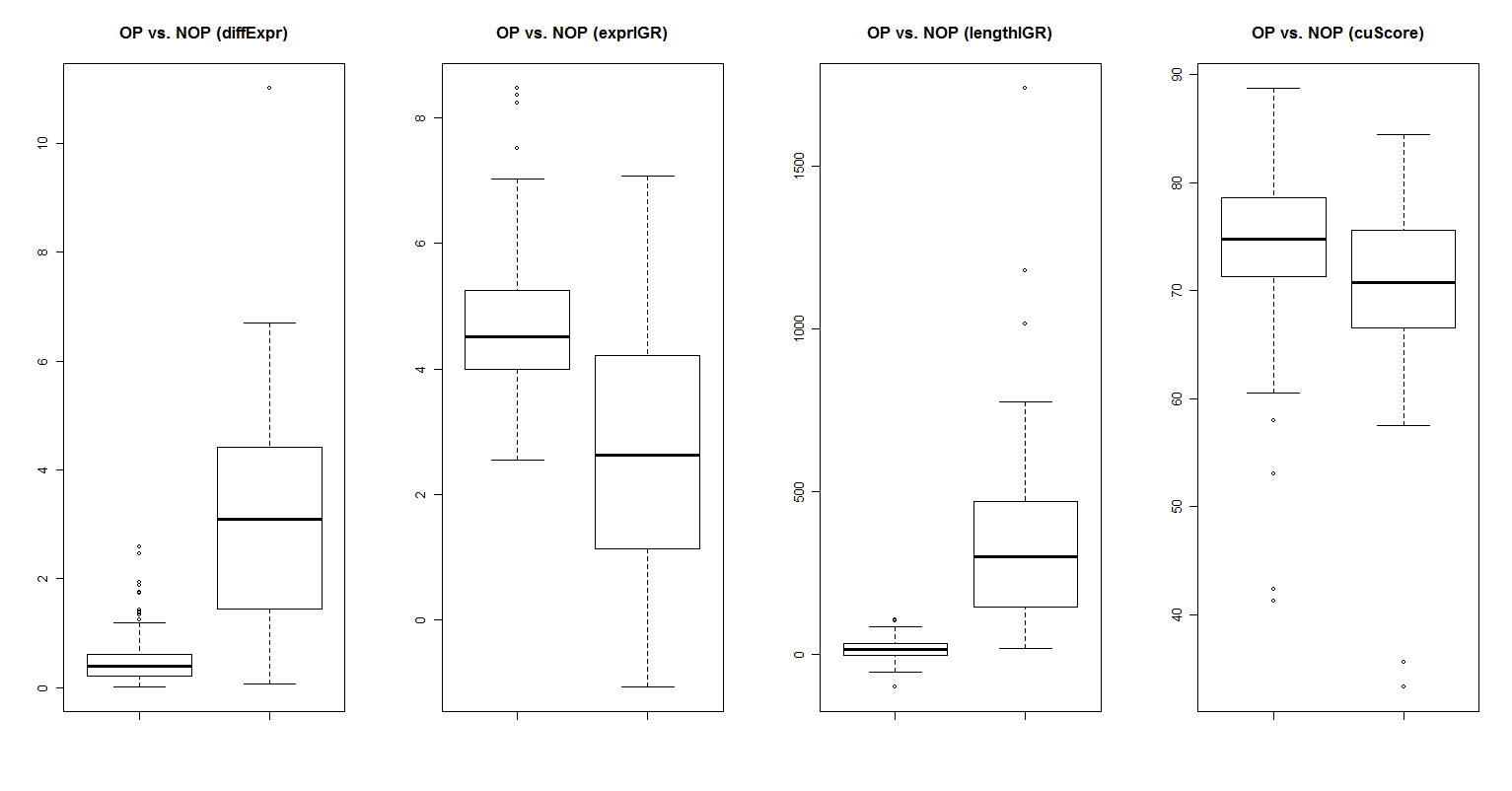


Figure 2 - Box plots showing the distribution of feature values for OPs and NOPs in PG2.


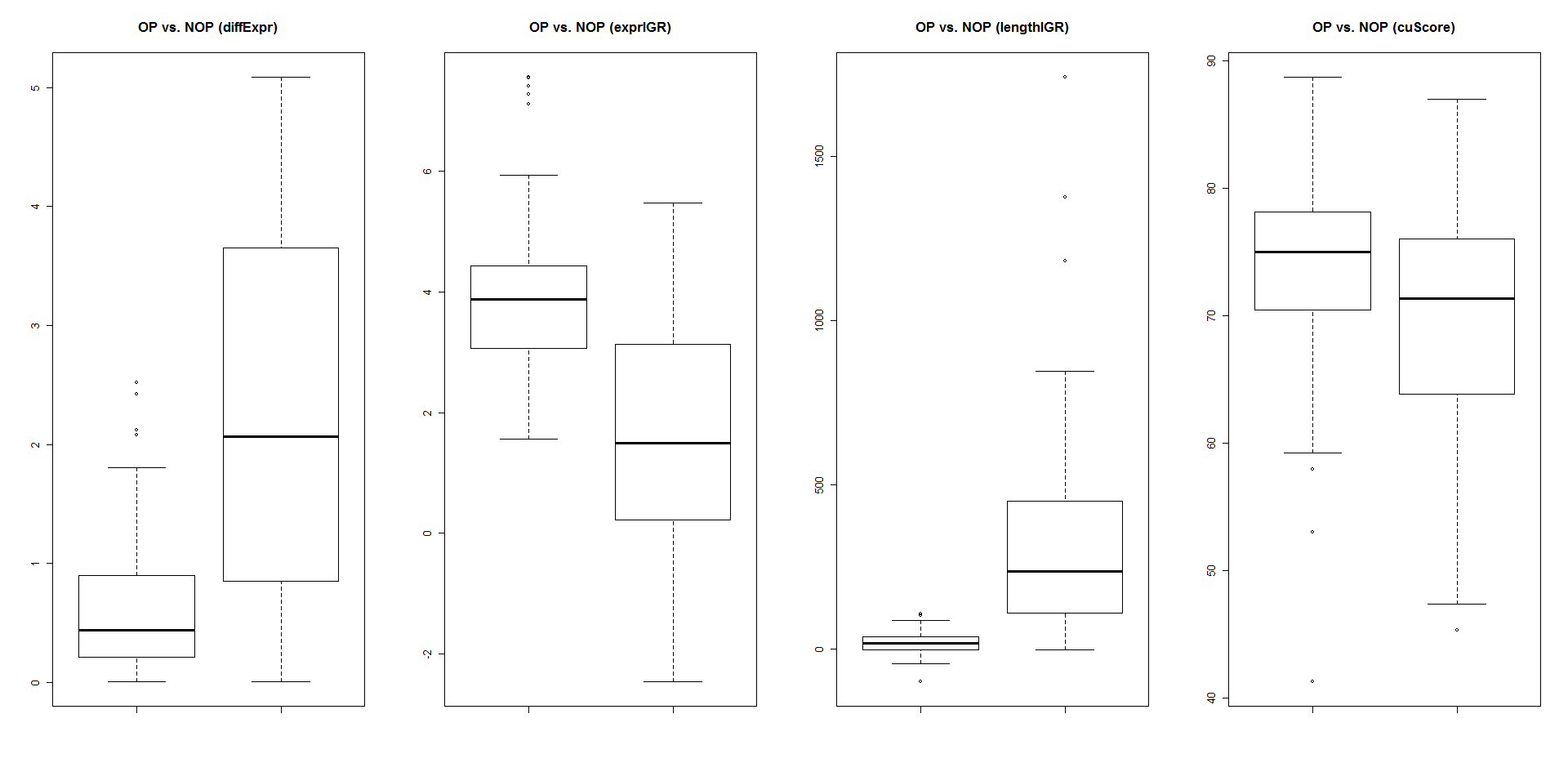


Figure 3 - Box plots showing the feature value distributions for OPs and NOPs in PG3.


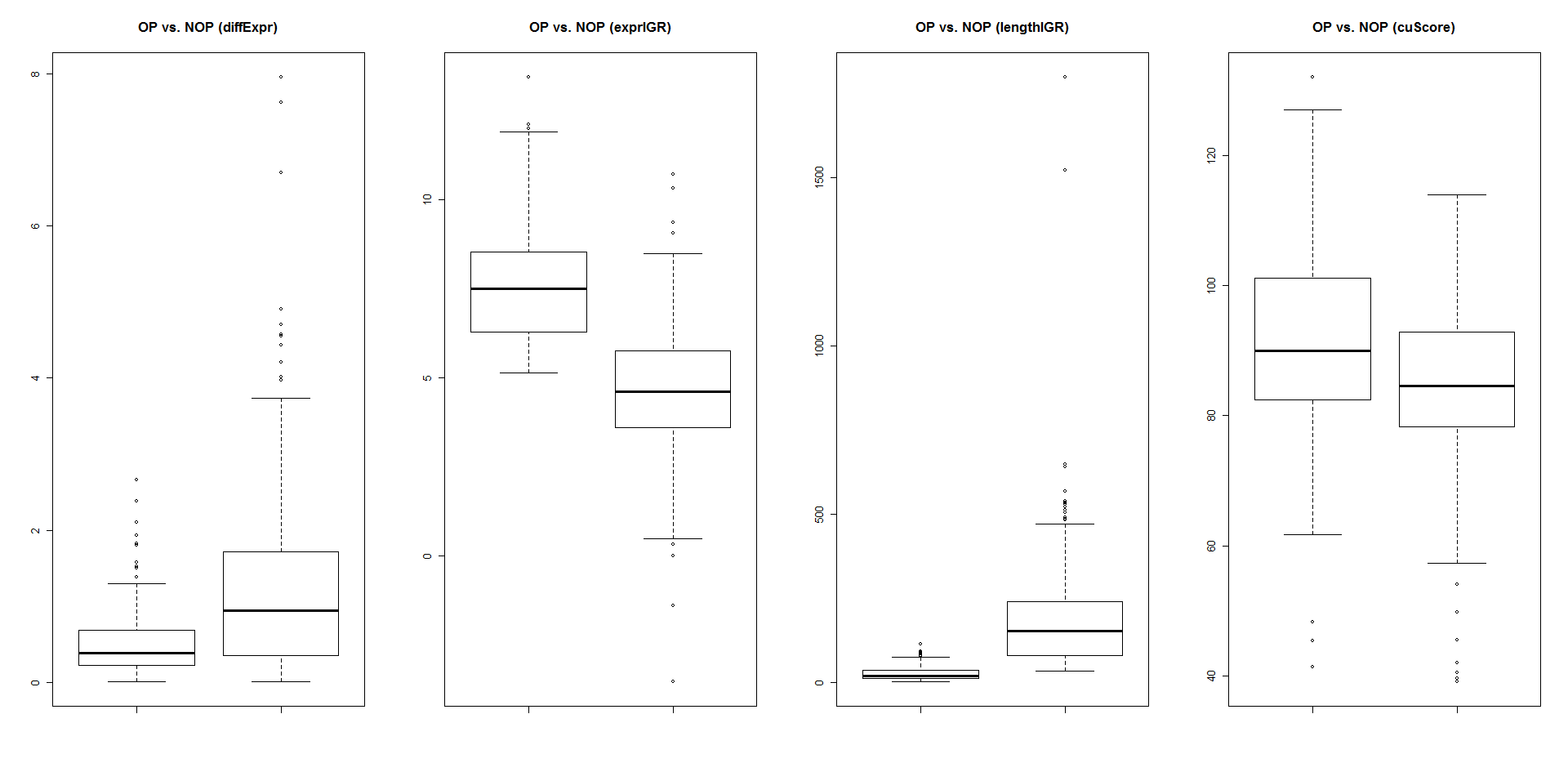


Figure 4 - Box plots showing the feature value distributions for OPs and NOPs in HS_2336.

# 4 - The RPKM function

$$RPKM(x)= \frac{{10}^{6}C}{NL/{10}^{3}}$$

Given RPKM to be the expression of gene *x*, *C* represented the number of reads that uniquely aligned to gene *x*, *N* represented the total number of reads that uniquely aligned to all genes, and *L* represented the number of bases on gene *x*.

# 5 - Details about the ROC curves and the input importance bar plots

PG1 (All features)


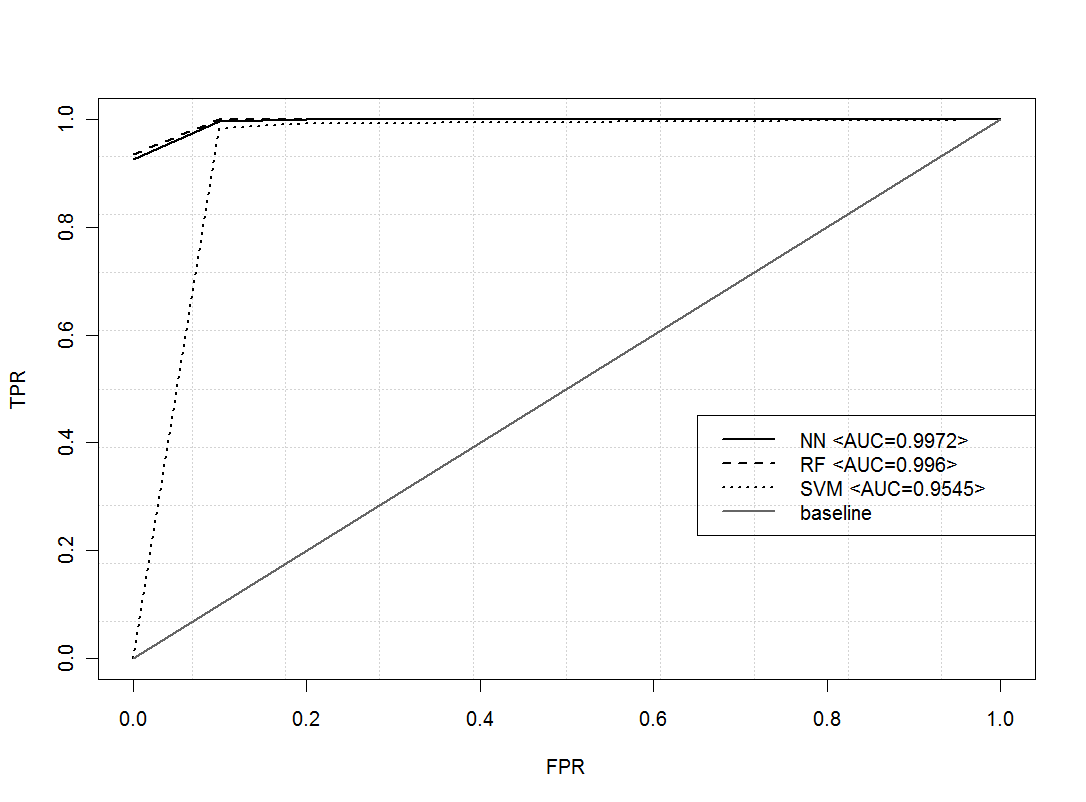


Figure 5 – Roc curves for “PG1”.


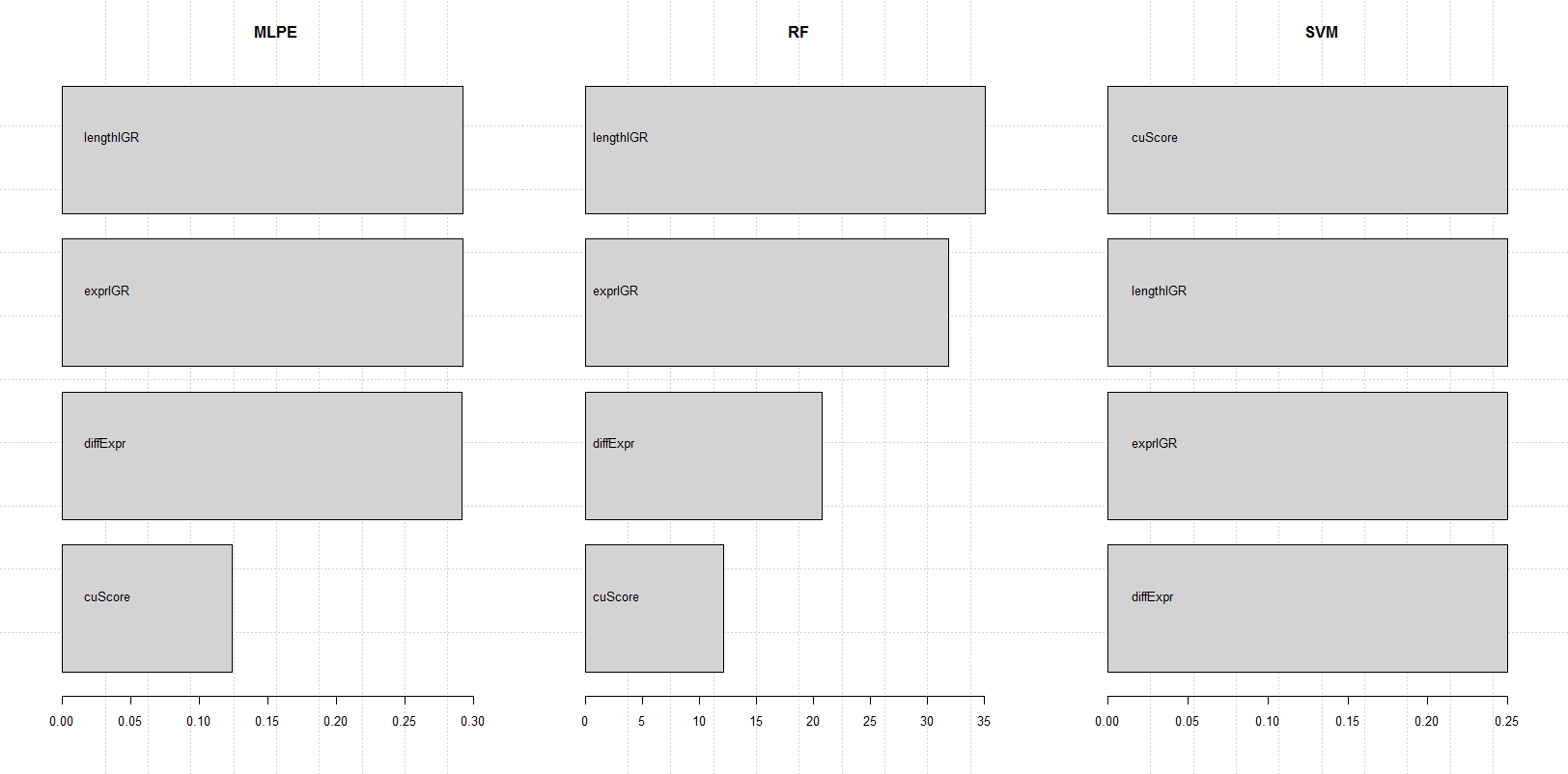


Figure 6 - Bar plots with the 1-D input importance”PG1”.

**Cross-validation**

**NN**

**AUC**

0.9966356 0.9974120 0.9994824 0.9992236 0.9989648 0.9981884 0.9974120 0.9956004 0.9956004 0.9937888

- M: 0.997230848861284
- SD: 0.00184234798483747

**ACC**

97.69231 98.46154 98.46154 98.46154 98.46154 98.46154 98.46154 96.15385 97.69231 95.38462

- M: 97.7692307692308
- SD: 1.11472128816842

pred

target NOP OP

NOP 440 20

OP 9 831

- "TPR: 0.989285714285714"
- "Precision: 0.976498237367803"
- "FPR: 0.689655172413793"
- "ACC: 0.977692307692308"
- "ER: 0.0223076923076923"

**RF**

**AUC**

0.9888393 0.9910714 1.0000000 0.9977679 1.0000000 1.0000000 1.0000000 1.0000000 0.9977679 0.9843750

- M: 0.995982142857143
- SD: 0.00574412472191035

**ACC**

95.45455 97.72727 100.00000 95.45455 97.72727 100.00000 95.45455 95.45455 95.45455 97.72727

- M: 97.0454545454545
- SD: 1.87107409624674

pred

target NOP OP

NOP 148 12

OP 1 279

- "TPR: 0.996428571428571"
- "Precision: 0.958762886597938"
- "FPR: 0.923076923076923"
- "ACC: 0.970454545454545"
- "ER: 0.0295454545454545"

**SVM**

**AUC**

0.9446170 0.9565217 0.9565217 0.9614389 0.9673913 0.9554865 0.9505694 0.9456522 0.9505694 0.9565217

- M: 0.954528985507246
- SD: 0.00694757826147719

**ACC**

95.38462 96.92308 96.92308 96.92308 97.69231 96.15385 96.15385 96.15385 96.15385 96.92308

- M: 96.5384615384615
- SD: 0.65372045046061

pred

target NOP OP

NOP 422 38

OP 7 833

- "TPR: 0.991666666666667"
- "Precision: 0.956371986222732"
- "FPR: 0.844444444444444"
- "ACC: 0.965384615384615"
- "ER: 0.0346153846153846"

**TEST-set**

**NN**

- AUC: 0.9864865
- ACC: 0.982456140350877
- ER: 0.0175438596491228

**RF**

- AUC: 1
- ACC: 0.982456140350877
- ER: 0.0175438596491228

**SVM**

- AUC: 0.9364865
- ACC: 0.947368421052632
- ER: 0.0526315789473684

PG1 – Genomic properties


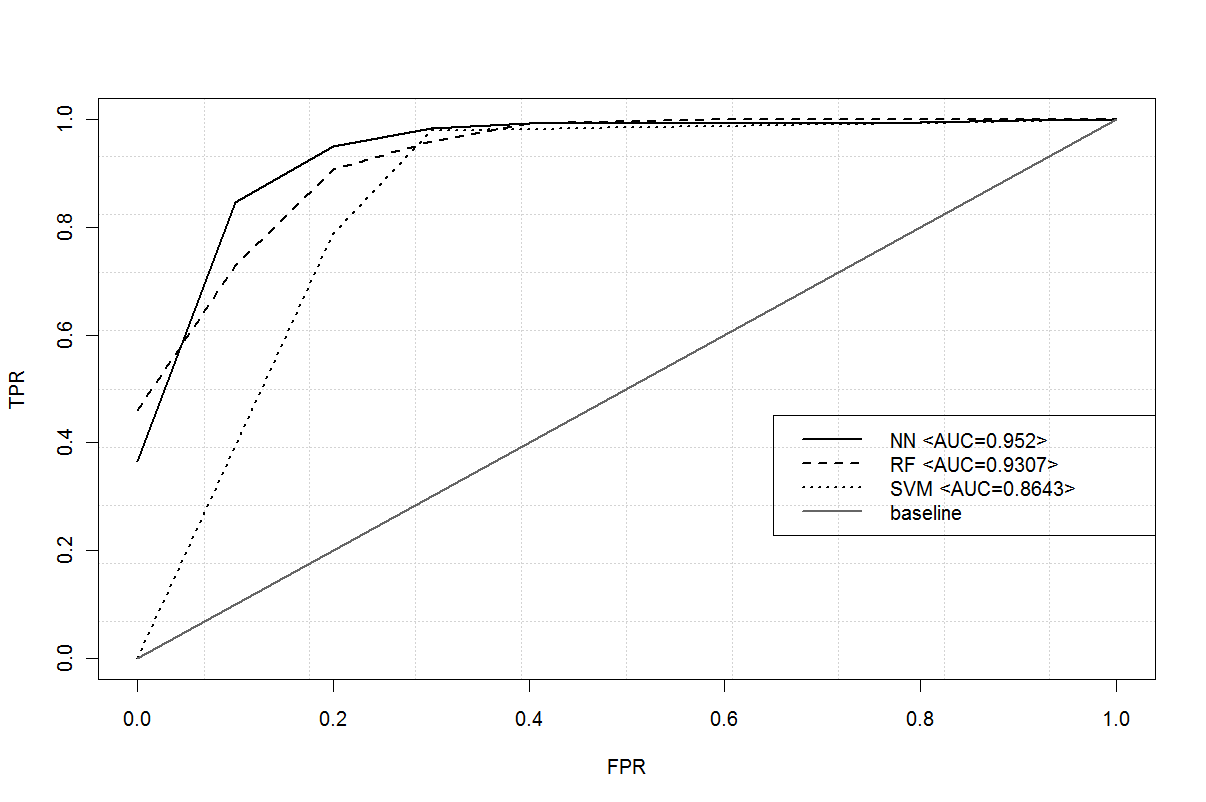


**Figure 7 – Roc curves achieved with the two genomic features “PG1”.**

**Cross-validation**

**NN**

**AUC**

0.9637681 0.9629917 0.9642857 0.9580745 0.9376294 0.9431936 0.9673913 0.9478520 0.9443582 0.9303830

- M: 0.951992753623188
- SD: 0.0129572003833807

**ACC**

91.53846 90.76923 89.23077 90.76923 89.23077 89.23077 90.00000 88.46154 87.69231 90.76923

- M: 89.7692307692308
- SD: 1.20540095113263

pred

target NOP OP

NOP 373 87

OP 46 794

- "TPR: 0.945238095238095"
- "Precision: 0.901248581157775"
- "FPR: 0.654135338345865"
- "ACC: 0.897692307692308"
- "ER: 0.102307692307692"

**RF**

**AUC**

0.9363839 0.8962054 0.8872768 0.9866071 0.9151786 0.9419643 0.9430804 0.9084821 0.9419643 0.9497768

- M: 0.930691964285714
- SD: 0.0293236425808215

**ACC**

86.36364 88.63636 86.36364 93.18182 88.63636 90.90909 88.63636 84.09091 90.90909 88.63636

- M: 88.6363636363636
- SD: 2.62431940540739

pred

target NOP OP

NOP 125 35

OP 15 265

- "TPR: 0.946428571428571"
- "Precision: 0.883333333333333"
- "FPR: 0.7"
- "ACC: 0.886363636363636"
- "ER: 0.113636363636364"

**SVM**

**AUC**

0.8744824 0.8793996 0.8636128 0.8467909 0.8576605 0.8695652 0.8853520 0.8636128 0.8457557 0.8566253

- M: 0.864285714285714
- SD: 0.0131528382934547

**ACC**

90.76923 90.76923 90.00000 88.46154 89.23077 90.76923 91.53846 90.00000 87.69231 88.46154

- M: 89.7692307692308
- SD: 1.25876284191114

pred

target NOP OP

NOP 345 115

OP 18 822

- "TPR: 0.978571428571429"
- "Precision: 0.87726787620064"
- "FPR: 0.864661654135338"
- "ACC: 0.897692307692308"
- "ER: 0.102307692307692"

**TEST-set**

**NN**

- AUC: 0.995946
- ACC: 0.947368421052632
- ER: 0.0526315789473684

**RF**

- AUC: 0.995946
- ACC: 0.964912280701754
- ER: 0.0350877192982456

**SVM**

- AUC: 0.9364865
- ACC: 0.947368421052632
- ER: 0.0526315789473684

PG1 – Transcriptomic properties


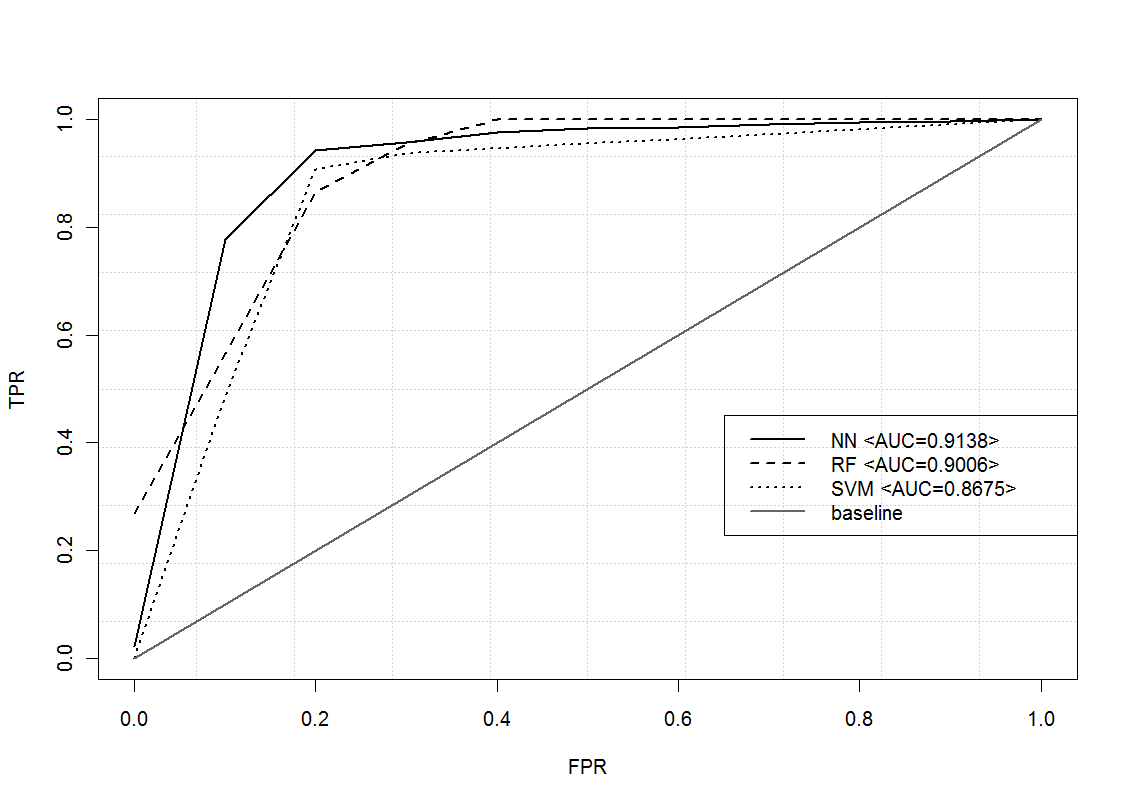


**Figure 8 – Roc curves achieved with the two transcriptomic features “PG1”.**

**Cross-validation**

**NN**

**AUC**

0.9153727 0.8990683 0.9171843 0.9332298 0.8837992 0.9101967 0.9327122 0.9329710 0.9026915 0.9104555

- M: 0.913768115942029
- SD: 0.016266099274964

**ACC**

89.23077 89.23077 90.00000 90.76923 87.69231 88.46154 89.23077 90.76923 88.46154 88.46154

- M: 89.2307692307692
- SD: 1.02564102564103

pred

target NOP OP

NOP 374 86

OP 54 786

- TPR: 0.935714285714286
- Precision: 0.90137614678899
- FPR: 0.614285714285714
- ACC: 0.892307692307692
- ER: 0.107692307692308

**RF**

**AUC**

0.8962054 0.8861607 0.8303571 0.9553571 0.9397321 0.8493304 0.8549107 0.9040179 0.9553571 0.9341518

- M: 0.900558035714286
- SD: 0.0453913261410072

**ACC**

84.09091 77.27273 88.63636 93.18182 90.90909 84.09091 81.81818 88.63636 90.90909 90.90909

- M: 87.0454545454545
- SD: 5.0308962775406

pred

target NOP OP

NOP 123 37

OP 20 260

- TPR: 0.928571428571429
- Precision: 0.875420875420875
- FPR: 0.649122807017544
- ACC: 0.870454545454545
- ER: 0.129545454545455

**SVM**

**AUC**

0.8724120 0.8377329 0.8605072 0.8496377 0.8783644 0.8881988 0.8832816 0.8615424 0.8654244 0.8783644

- M: 0.867546583850932
- SD: 0.0157462985888261

**ACC**

89.23077 85.38462 87.69231 86.92308 90.00000 90.00000 90.00000 88.46154 87.69231 90.00000

- M: 88.5384615384615
- SD: 1.59922729533463

pred

target NOP OP

NOP 371 89

OP 60 780

- TPR: 0.928571428571429
- Precision: 0.897583429228999
- FPR: 0.597315436241611
- ACC: 0.885384615384615
- ER: 0.114615384615385

**TEST-set**

**NN**

- AUC: 0.8513514
- ACC: 0.842105263157895
- ER: 0.157894736842105

**RF**

- AUC: 0.8804054
- ACC: 0.842105263157895
- ER: 0.157894736842105

**SVM**

- AUC: 0.797973
- ACC: 0.842105263157895
- ER: 0.157894736842105

PG1 – Genomic properties + ExprIGR


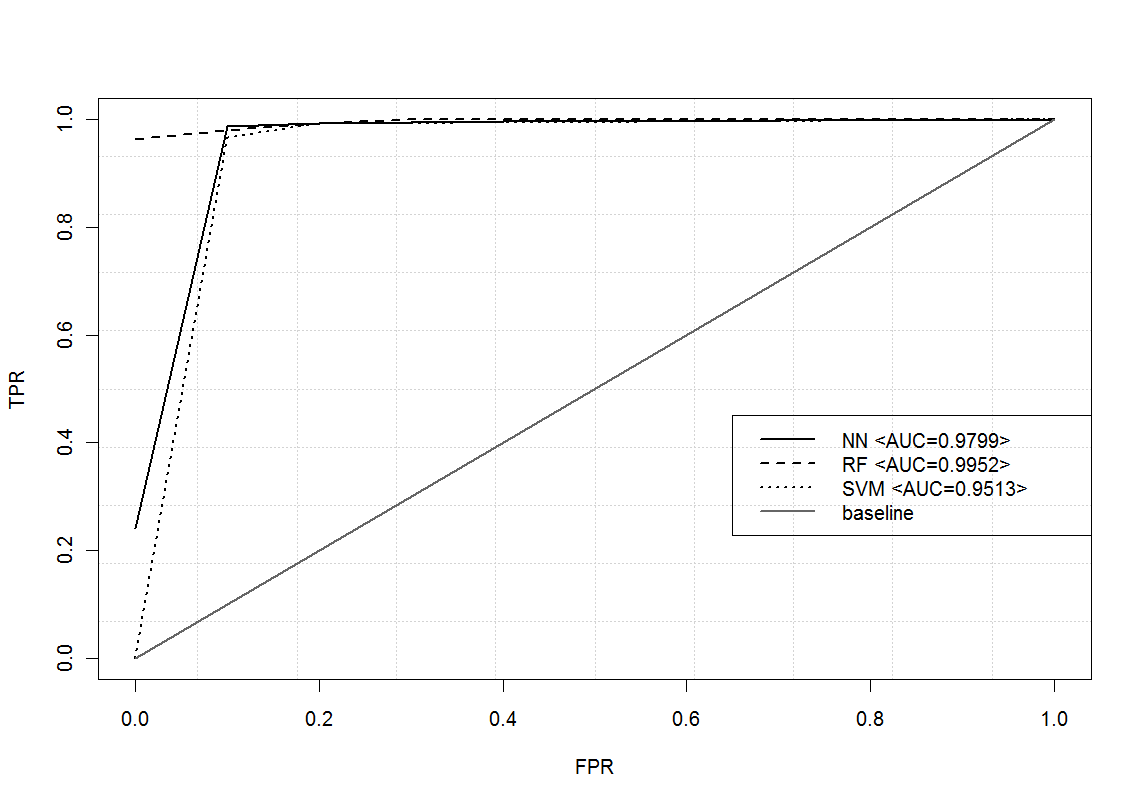


**Figure 9 – Roc curves achieved combining the first transcriptomic feature with the two genomic features “PG1”.**

**Cross-validation**

**NN**

**AUC**

0.9785197 0.9901656 0.9956004 0.9745083 0.9663561 0.9658385 0.9966356 0.9798137 0.9774845 0.9736025

- M: 0.97985248447205
- SD: 0.0110029475596779

**ACC**

96.15385 96.15385 96.92308 95.38462 96.15385 96.15385 96.15385 96.15385 96.92308 97.69231

- M: 96.3846153846154
- SD: 0.633286617191201

pred

target NOP OP

NOP 434 26

OP 21 819

- TPR: 0.975
- Precision: 0.96923076923077
- FPR: 0.553191489361702
- ACC: 0.963846153846154
- ER: 0.0361538461538462

**RF**

**AUC**

1.0000000 1.0000000 0.9966518 0.9843750 0.9955357 1.0000000 1.0000000 0.9866071 0.9977679 0.9910714

- M: 0.995200892857143
- SD: 0.00585981786875086

**ACC**

100.00000 100.00000 95.45455 88.63636 97.72727 100.00000 97.72727 93.18182 95.45455 90.90909

- M: 95.909090909091
- SD: 3.97997743532233

pred

target NOP OP

NOP 146 14

OP 4 276

- TPR: 0.985714285714286
- Precision: 0.951724137931034
- FPR: 0.777777777777778
- ACC: 0.95909090909091
- ER: 0.0409090909090909

**SVM**

AUC

0.9456522 0.9218427 0.9673913 0.9565217 0.9505694 0.9614389 0.9565217 0.9456522 0.9505694 0.9565217

- M: 0.951268115942029
- SD: 0.0123653475164587

ACC

96.15385 93.07692 97.69231 96.92308 96.15385 96.92308 96.92308 96.15385 96.15385 96.92308

- M: 96.3076923076923
- SD: 1.24563669758883

pred

target NOP OP

NOP 419 41

OP 7 833

- TPR: 0.991666666666667
- Precision: 0.953089244851259
- FPR: 0.854166666666667
- ACC: 0.963076923076923
- ER: 0.0369230769230769

**TEST-set**

**NN**

- AUC: 0.9594595
- ACC: 0.947368421052632
- ER: 0.0526315789473684

**RF**

- AUC: 0.9614865
- ACC: 0.947368421052632
- ER: 0.0526315789473684

**SVM**

- AUC: 0.925
- ACC: 0.947368421052632
- ER: 0.0526315789473684

PG1 – Genomic properties + DiffExpr


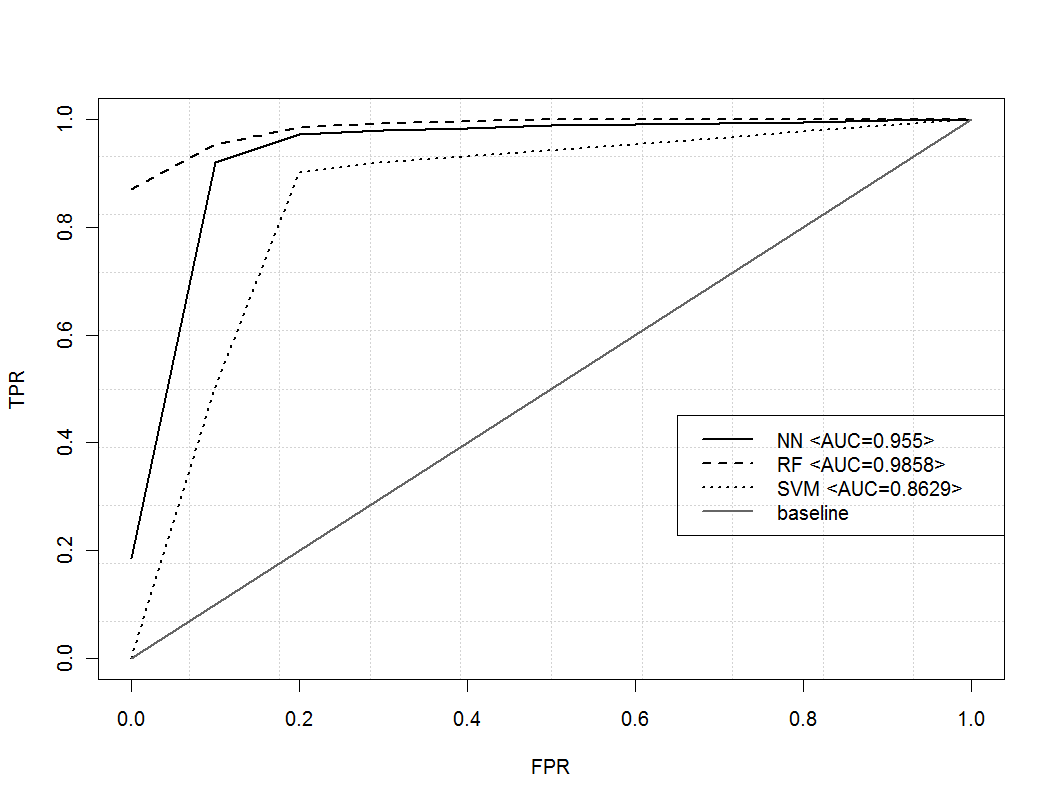


**Figure 10 – Roc curves achieved combining the second transcriptomic feature with the two genomic features “PG1”.**

**Cross-validation**

**NN**

**AUC**

0.9702381 0.9257246 0.9818841 0.9535455 0.9631211 0.9628623 0.9434524 0.9372412 0.9697205 0.9421584

- M: 0.954994824016563
- SD: 0.0175505786243131

**ACC**

95.38462 88.46154 93.07692 92.30769 92.30769 91.53846 92.30769 91.53846 92.30769 90.00000

- M: 91.9230769230769
- SD: 1.8221372312246

pred

target NOP OP

NOP 405 55

OP 50 790

- TPR: 0.94047619047619
- Precision: 0.93491124260355
- FPR: 0.523809523809524
- ACC: 0.919230769230769
- ER: 0.0807692307692308

**RF**

**AUC**

0.9732143 0.9799107 0.9754464 0.9709821 0.9888393 0.9910714 0.9977679 0.9977679 0.9866071 0.9966518

- M: 0.985825892857143
- SD: 0.0103373093229185

**ACC**

88.63636 93.18182 93.18182 95.45455 90.90909 95.45455 97.72727 97.72727 95.45455 97.72727

- M: 94.5454545454545
- SD: 3.06794798959342

pred

target NOP OP

NOP 144 16

OP 8 272

- TPR: 0.971428571428571
- Precision: 0.944444444444444
- FPR: 0.666666666666667
- ACC: 0.945454545454545
- ER: 0.0545454545454545

**SVM**

**AUC**

0.8713768 0.8713768 0.8486025 0.8486025 0.8555901 0.8545549 0.8703416 0.8762940 0.8605072 0.8713768

- M: 0.86286231884058
- SD: 0.0104773363698892

**ACC**

88.46154 88.46154 86.15385 86.15385 87.69231 86.92308 87.69231 88.46154 87.69231 88.46154

- M: 87.6153846153846
- SD: 0.92093769210605

pred

target NOP OP

NOP 376 84

OP 77 763

- TPR: 0.908333333333333
- Precision: 0.900826446280992
- FPR: 0.521739130434783
- ACC: 0.876153846153846
- ER: 0.123846153846154

**TEST-set**

**NN**

- AUC: 0.9371622
- ACC: 0.912280701754386
- ER: 0.087719298245614

**RF**

- AUC: 0.9466216
- ACC: 0.947368421052632
- ER: 0.0526315789473684

**SVM**

- AUC: 0.9
- ACC: 0.929824561403509
- ER: 0.0701754385964912

PG2 (All featutes)


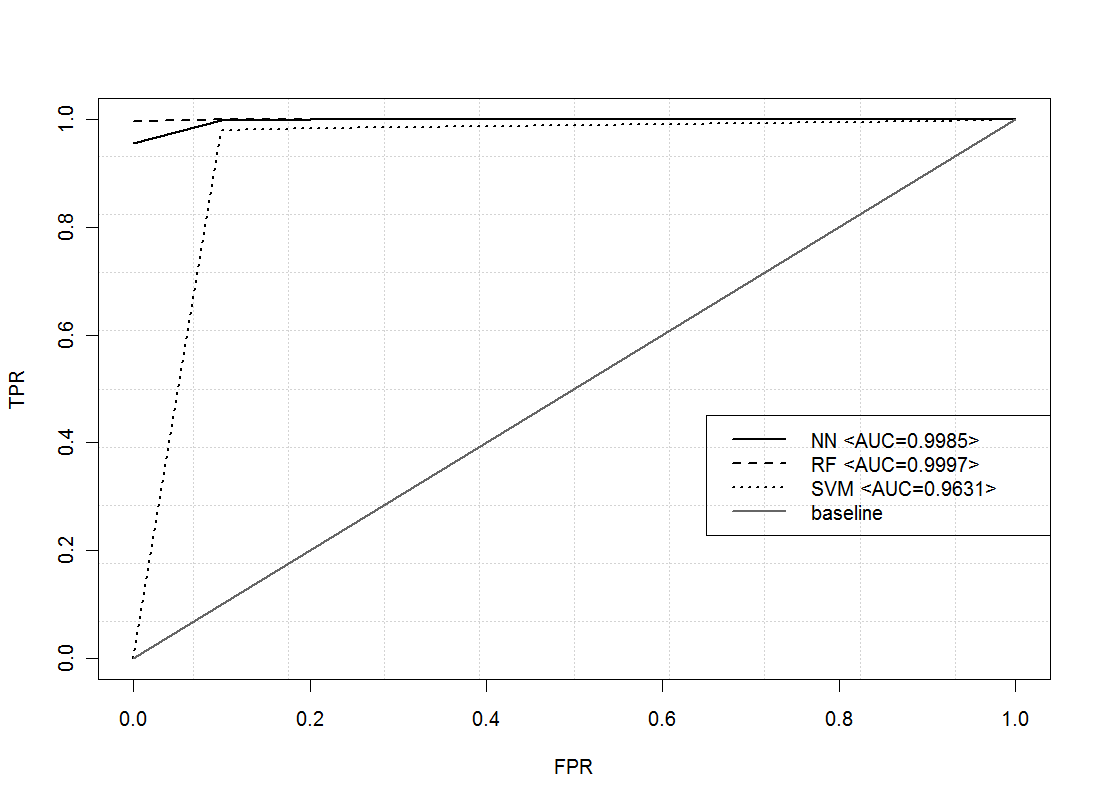


**Figure 11 – Roc curves for “PG2”.**


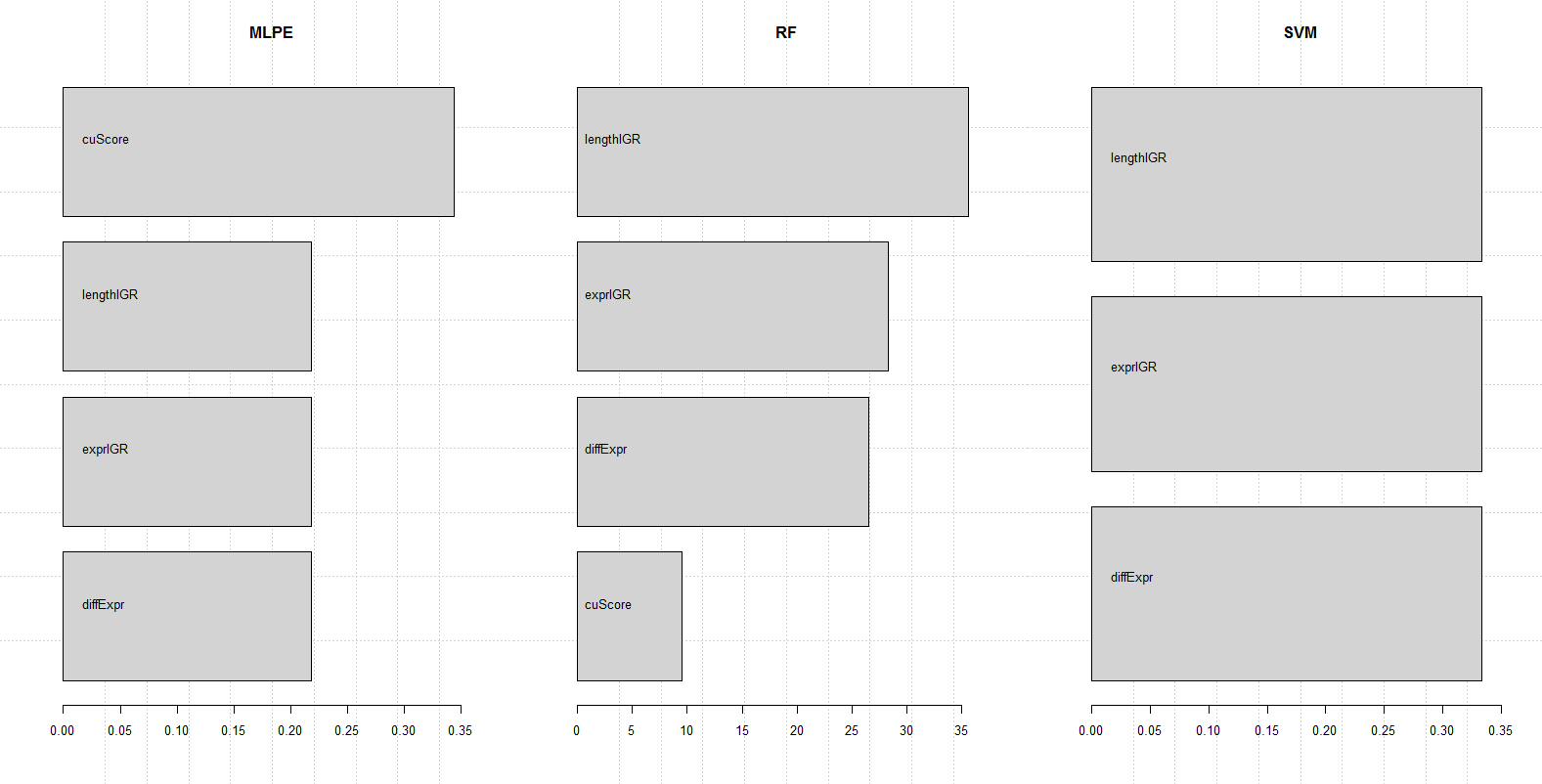


Figure 12 - Bar plots with the 1-D input importance”PG2”.

**Cross-validation**

**NN**

**AUC**

0.9996857 0.9981144 0.9993715 0.9996857 0.9984287 0.9993715 0.9984287 0.9956003 0.9974859 0.9990572

- M: 0.998522941546197
- SD: 0.00125750743175551

**ACC**

99.18699 98.37398 99.18699 99.18699 98.37398 98.37398 99.18699 96.74797 97.56098 99.18699

- M: 98.5365853658537
- SD: 0.839671186169629

pred

target NOP OP

NOP 353 17

OP 1 859

- TPR: 0.998837209302326
- Precision: 0.980593607305936
- FPR: 0.944444444444444
- ACC: 0.985365853658537
- ER: 0.0146341463414634

**RF**

**AUC**

1.0000000 1.0000000 1.0000000 0.9973475 1.0000000 1.0000000 1.0000000 1.0000000 1.0000000 1.0000000

- M: 0.99973474801061
- SD: 0.00083880044036298

**ACC**

100.00000 100.00000 97.61905 95.23810 100.00000 100.00000 97.61905 100.00000 100.00000 100.00000

- M: 99.047619047619
- SD: 1.66477594947643

pred

target NOP OP

NOP 128 2

OP 2 288

- TPR: 0.993103448275862
- Precision: 0.993103448275862
- FPR: 0.5
- ACC: 0.99047619047619
- ER: 0.00952380952380952

**SVM**

**AUC**

0.9536455 0.9806725 0.9478316 0.9497172 0.9671590 0.9748586 0.9671590 0.9613451 0.9748586 0.9536455

- M: 0.96308925204274
- SD: 0.0116062868674769

**ACC**

96.74797 98.37398 95.93496 95.12195 97.56098 97.56098 97.56098 96.74797 97.56098 96.74797

- M: 96.9918699186992
- SD: 0.942684397340167

pred

target NOP OP

NOP 350 20

OP 17 843

- TPR: 0.980232558139535
- Precision: 0.976825028968714
- FPR: 0.540540540540541
- ACC: 0.969918699186992
- ER: 0.0300813008130081

**TEST-set**

**NN**

- AUC: 1
- ACC: 0.981481481481482
- ER: 0.0185185185185185

**RF**

- AUC**:** 1
- ACC: 0.981481481481482
- ER: 0.0185185185185185

**SVM**

- AUC: 0.96875
- ACC: 0.981481481481482
- ER: 0.0185185185185185

PG2 – Genomic properties


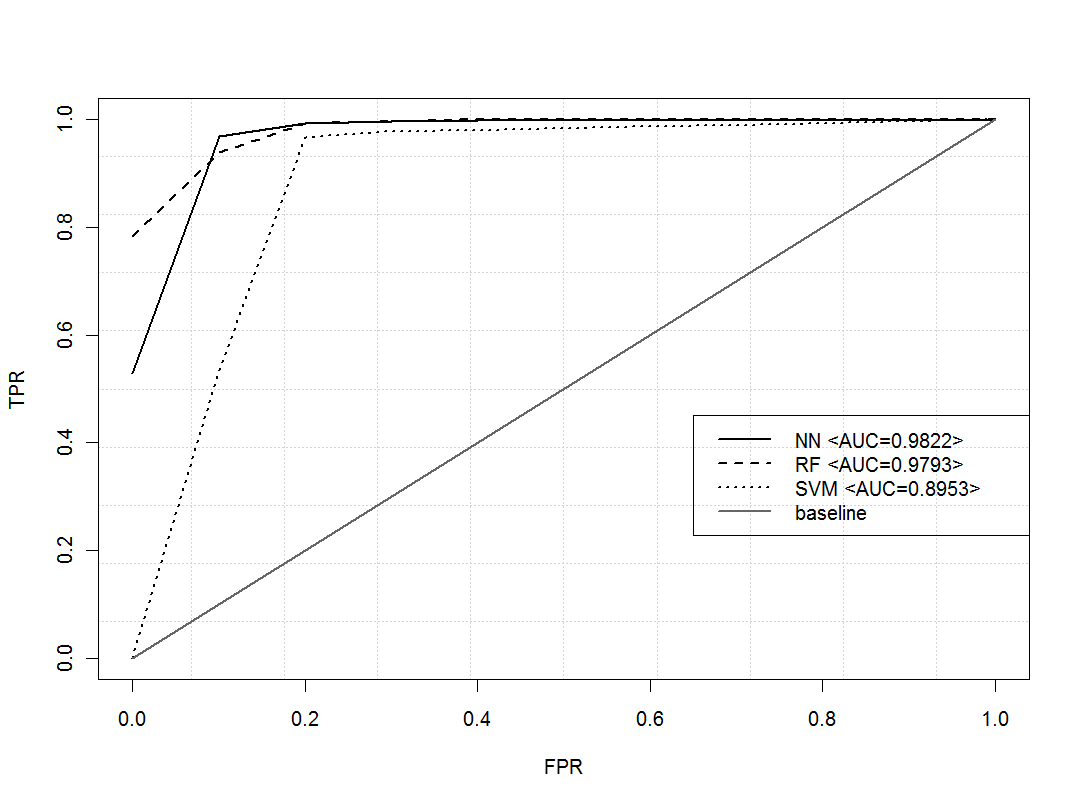


**Figure 13 – Roc curves achieved with the two genomic features “PG2”.**

**Cross-validation**

**NN**

**AUC**

0.9849151 0.9802011 0.9728158 0.9792583 0.9899434 0.9882150 0.9949717 0.9940289 0.9725016 0.9648020

- M: 0.982165304839723
- SD: 0.0100222046501951

**ACC**

93.49593 96.74797 93.49593 93.49593 95.12195 95.93496 95.12195 96.74797 95.93496 96.74797

- M: 95.2845528455285
- SD: 1.37117730522206

pred

target NOP OP

NOP 340 30

OP 28 832

- TPR: 0.967441860465116
- Precision: 0.965197215777262
- FPR: 0.517241379310345
- ACC: 0.952845528455285
- ER: 0.0471544715447154

**RF**

**AUC**

0.9535809 0.9973475 0.9814324 0.9867374 0.9933687 0.9893899 0.9893899 0.9920424 0.9098143 1.0000000

- M: 0.979310344827586
- SD: 0.0276238719041843

**ACC**

92.85714 97.61905 97.61905 90.47619 95.23810 97.61905 95.23810 95.23810 88.09524 97.61905

- M: 94.7619047619048
- SD: 3.32955189895286

pred

target NOP OP

NOP 118 12

OP 10 280

- TPR: 0.96551724137931
- Precision: 0.95890410958904
- FPR: 0.545454545454545
- ACC: 0.947619047619048
- ER: 0.0523809523809524

**SVM**

**AUC**

0.8879635 0.9131050 0.9131050 0.8821496 0.8879635 0.8879635 0.8821496 0.8860779 0.9131050 0.8995915

- M: 0.89531741043369
- SD: 0.0131734056123811

**ACC**

91.86992 94.30894 94.30894 91.05691 91.86992 91.86992 91.05691 92.68293 94.30894 93.49593

- M: 92.6829268292683
- SD: 1.32763671695565

pred

target NOP OP

NOP 302 68

OP 22 838

- TPR: 0.974418604651163
- Precision: 0.924944812362031
- FPR: 0.755555555555556
- ACC: 0.926829268292683
- ER: 0.073170731707317

**TEST-set**

**NN**

- AUC: 1
- ACC: 0.981481481481482
- ER: 0.0185185185185185

**RF**

- AUC: 0.9983553
- ACC: 0.962962962962963
- ER: 0.037037037037037

**SVM**

- AUC: 0.96875
- ACC: 0.981481481481482
- ER: 0.0185185185185185

PG2 – Transcriptomic properties


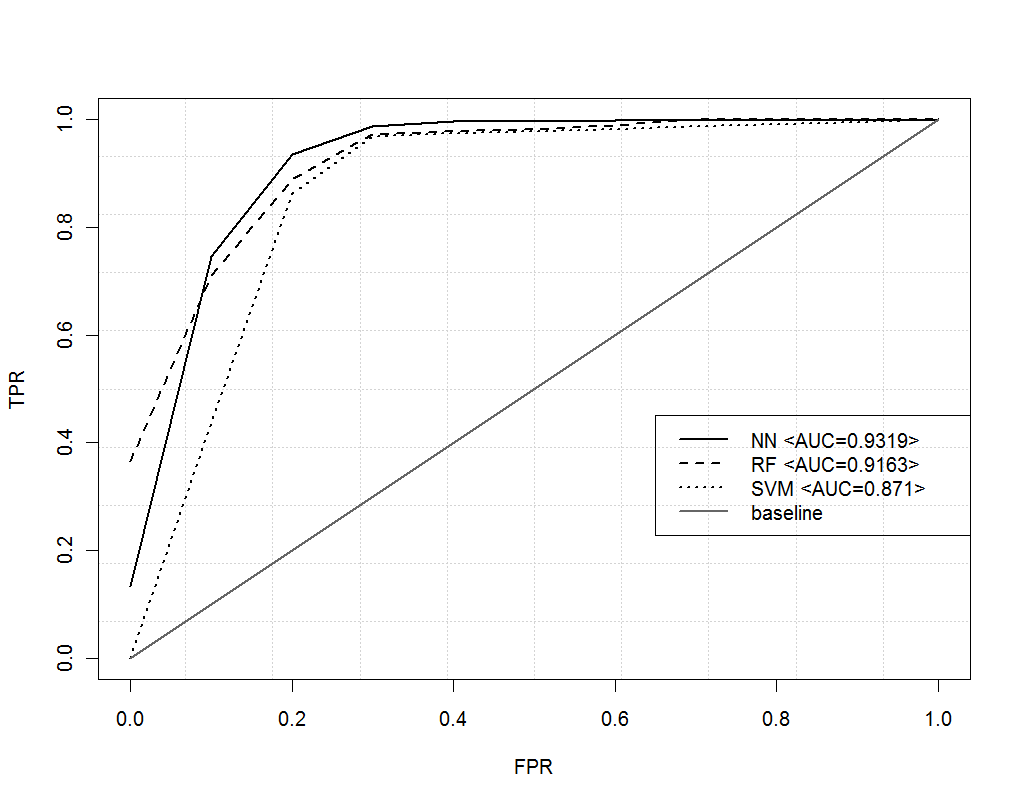


**Figure 14 – Roc curves achieved with the two transcriptomic features “PG2”.**

**Cross-validation**

**NN**

**AUC**

0.9384035 0.9440603 0.9428033 0.9343180 0.9261471 0.9374607 0.9206474 0.8999057 0.9349466 0.9406034

- M: 0.931929604022627
- SD: 0.0133923385096024

**ACC**

90.24390 91.05691 91.05691 88.61789 91.05691 91.86992 89.43089 88.61789 91.86992 89.43089

- M: 90.3252032520325
- SD: 1.23893002175203

pred

target NOP OP

NOP 290 80

OP 39 821

- TPR: 0.954651162790698
- Precision: 0.911209766925638
- FPR: 0.672268907563025
- ACC: 0.903252032520325
- ER: 0.0967479674796748

**RF**

**AUC**

1.0000000 0.8885942 0.8766578 0.9416446 0.9310345 0.9774536 0.9416446 0.8700265 0.8620690 0.8740053

- M: 0.91631299734748
- SD: 0.048827864983905

**ACC**

97.61905 83.33333 88.09524 83.33333 95.23810 90.47619 92.85714 92.85714 88.09524 88.09524

- M: 90
- SD: 4.73537583815121

pred

target NOP OP

NOP 105 25

OP 17 273

- TPR: 0.941379310344828
- Precision: 0.916107382550336
- FPR: 0.595238095238095
- ACC: 0.9
- ER: 0.1

**SVM**

**AUC**

0.8802640 0.8879635 0.8551226 0.8744500 0.8590509 0.8802640 0.8744500 0.8416091 0.8821496 0.8744500

- M: 0.870977372721559
- SD: 0.0144457709221081

**ACC**

91.86992 91.86992 89.43089 91.05691 91.05691 91.86992 91.05691 88.61789 91.05691 91.05691

- M: 90.8943089430894
- SD: 1.07037494082013

pred

target NOP OP

NOP 287 83

OP 29 831

- TPR: 0.966279069767442
- Precision: 0.909190371991247
- FPR: 0.741071428571429
- ACC: 0.908943089430894
- ER: 0.0910569105691057

**TEST-set**

**NN**

- AUC: 0.9539474
- ACC: 0.925925925925926
- ER: 0.0740740740740741

**RF**

- AUC: 0.9901316
- ACC: 0.925925925925926
- ER: 0.0740740740740741

**SVM**

- AUC: 0.84375
- ACC: 0.907407407407407
- ER: 0.0925925925925926

PG2 – Genomic properties + ExprIGR


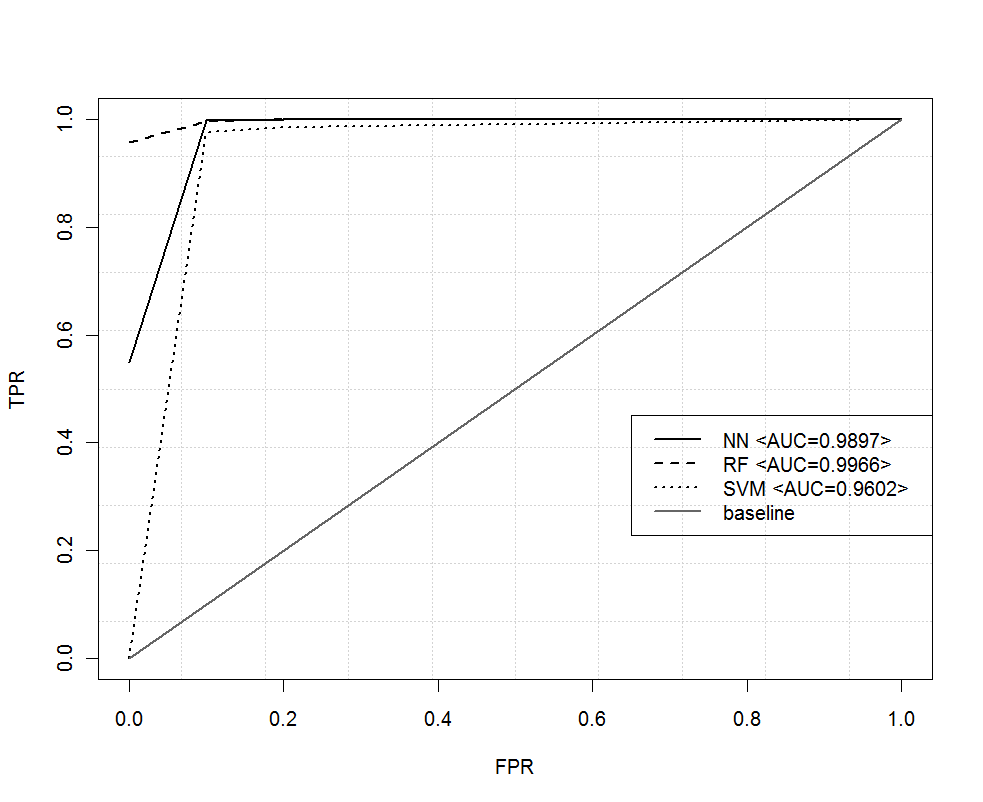


**Figure 15 – Roc curves achieved combining the first transcriptomic feature with the two genomic features “PG2”.**

**Cross-validation**

**NN**

**AUC**

0.9835009 0.9828724 0.9981144 0.9968573 0.9965431 0.9949717 0.9676304 0.9965431 0.9974859 0.9820867

- M: 0.989660590823382
- SD: 0.0101879641543343

**ACC**

95.93496 96.74797 98.37398 96.74797 97.56098 96.74797 96.74797 97.56098 98.37398 98.37398

- M: 97.3170731707317
- SD: 0.861260085749089

pred

target NOP OP

NOP 351 19

OP 14 846

- TPR: 0.983720930232558
- Precision: 0.978034682080925
- FPR: 0.575757575757576
- ACC: 0.973170731707317
- ER: 0.0268292682926829

**RF**

**AUC**

0.9920424 0.9973475 1.0000000 1.0000000 1.0000000 0.9946950 0.9946950 1.0000000 0.9946950 0.9920424

- M: 0.99655172413793
- SD: 0.00332006779054264

**ACC**

97.61905 97.61905 100.00000 95.23810 97.61905 95.23810 92.85714 100.00000 97.61905 97.61905

- M: 97.1428571428571
- SD: 2.18794424636353

pred

target NOP OP

NOP 120 10

OP 2 288

- TPR: 0.993103448275862
- Precision: 0.966442953020134
- FPR: 0.833333333333333
- ACC: 0.971428571428571
- ER: 0.0285714285714286

**SVM**

**AUC**

0.9343180 0.9613451 0.9671590 0.9478316 0.9671590 0.9671590 0.9613451 0.9613451 0.9671590 0.9671590

- M: 0.96019798868636
- SD: 0.0108981530019041

**ACC**

95.12195 96.74797 97.56098 95.93496 97.56098 97.56098 96.74797 96.74797 97.56098 97.56098

- M: 96.910569105691
- SD: 0.839671186169629

pred

target NOP OP

NOP 347 23

OP 15 845

- TPR: 0.982558139534884
- Precision: 0.973502304147465
- FPR: 0.605263157894737
- ACC: 0.96910569105691
- ER: 0.0308943089430894

**TEST-set**

**NN**

- AUC: 1
- ACC: 1"
- ER: 0

**RF**

- AUC: 1
- ACC: 1
- ER: 0

**SVM**

- AUC: 0.96875
- ACC: 0.981481481481482
- ER: 0.0185185185185185

PG2 – Genomic properties + DiffExpr


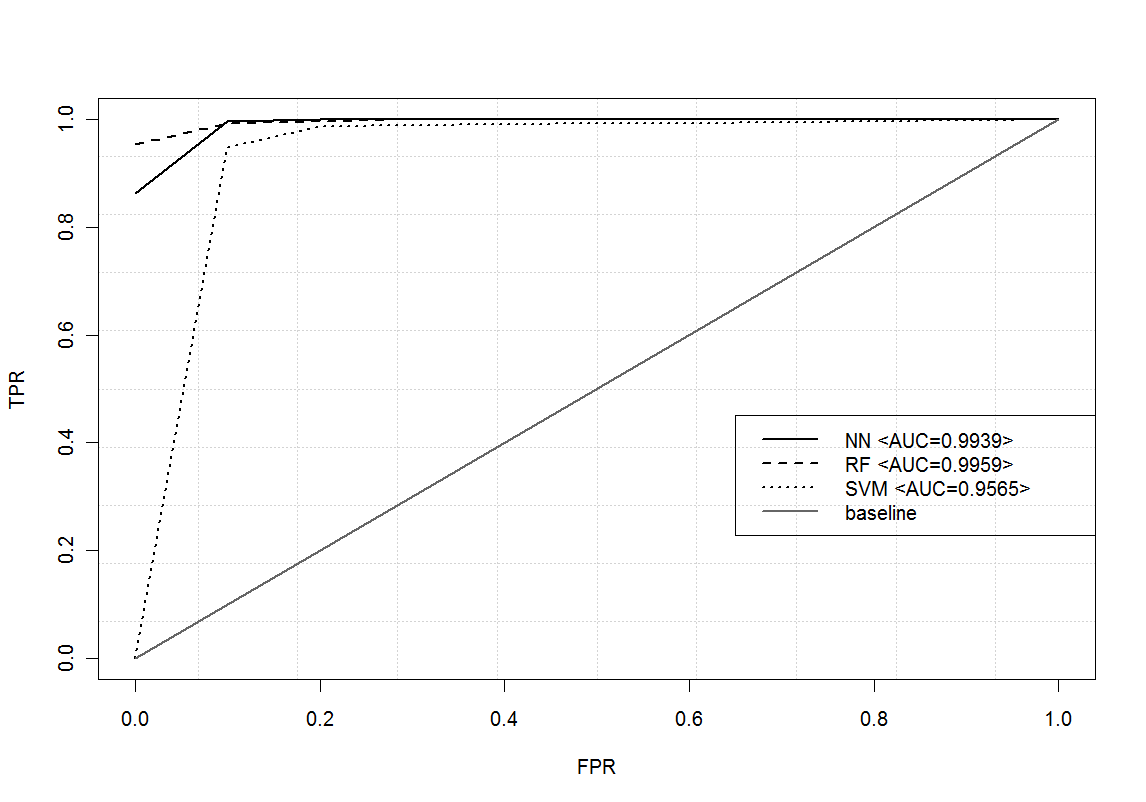


**Figure 16 – Roc curves achieved combining the second transcriptomic feature with the two genomic features “PG2”.**

**Cross-validation**

**NN**

**AUC**

0.9974859 0.9981144 0.9959145 0.9978001 0.9993715 0.9575739 0.9987429 0.9981144 0.9978001 0.9984287

- M: 0.993934632306725
- SD: 0.0128077124116599

**ACC**

96.74797 97.56098 95.93496 97.56098 97.56098 96.74797 98.37398 98.37398 96.74797 96.74797

- M: 97.2357723577236
- SD: 0.785440474048208

pred

target NOP OP

NOP 347 23

OP 11 849

- TPR: 0.987209302325581
- Precision: 0.97362385321101
- FPR: 0.676470588235294
- ACC: 0.972357723577236
- ER: 0.0276422764227642

**RF**

**AUC**

1.0000000 1.0000000 1.0000000 1.0000000 0.9973475 0.9946950 0.9920424 1.0000000 0.9774536 0.9973475

- M: 0.995888594164456
- SD: 0.00703042879612657

**ACC**

92.85714 97.61905 97.61905 100.00000 97.61905 92.85714 95.23810 100.00000 95.23810 90.47619

- M: 95.952380952381
- SD: 3.18450835678395

pred

target NOP OP

NOP 116 14

OP 3 287

- TPR: 0.989655172413793
- Precision: 0.953488372093023
- FPR: 0.823529411764706
- ACC: 0.95952380952381
- ER: 0.0404761904761905

**SVM**

**AUC**

0.9671590 0.9671590 0.9671590 0.9478316 0.9420176 0.9189189 0.9671590 0.9671590 0.9671590 0.9536455

- M: 0.956536769327467
- SD: 0.0162857892076246

**ACC**

97.56098 97.56098 97.56098 95.93496 95.12195 95.12195 97.56098 97.56098 97.56098 96.74797

- M: 96.829268292683
- SD: 1.04608450220156

pred

target NOP OP

NOP 343 27

OP 12 848

- TPR: 0.986046511627907
- Precision: 0.969142857142857
- FPR: 0.692307692307692
- ACC: 0.96829268292683
- ER: 0.0317073170731707

**TEST-set**

**NN**

- AUC: 0.9523026
- ACC: 0.962962962962963
- ER: 0.037037037037037

**RF**

- AUC: 0.9901316
- ACC: 0.962962962962963
- ER: 0.037037037037037

**SVM**

- AUC: 0.96875
- ACC: 0.981481481481482
- ER: 0.0185185185185185

PG3 (All featutes)


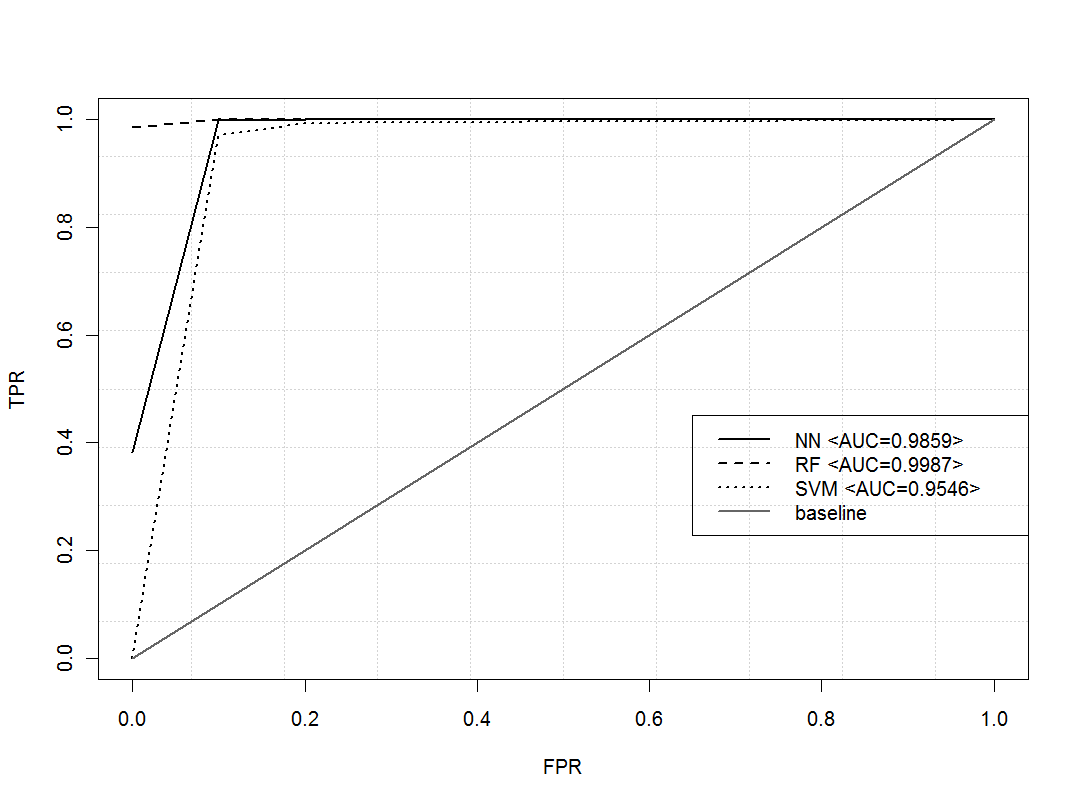


**Figure 17 – Roc curves for “PG3”.**


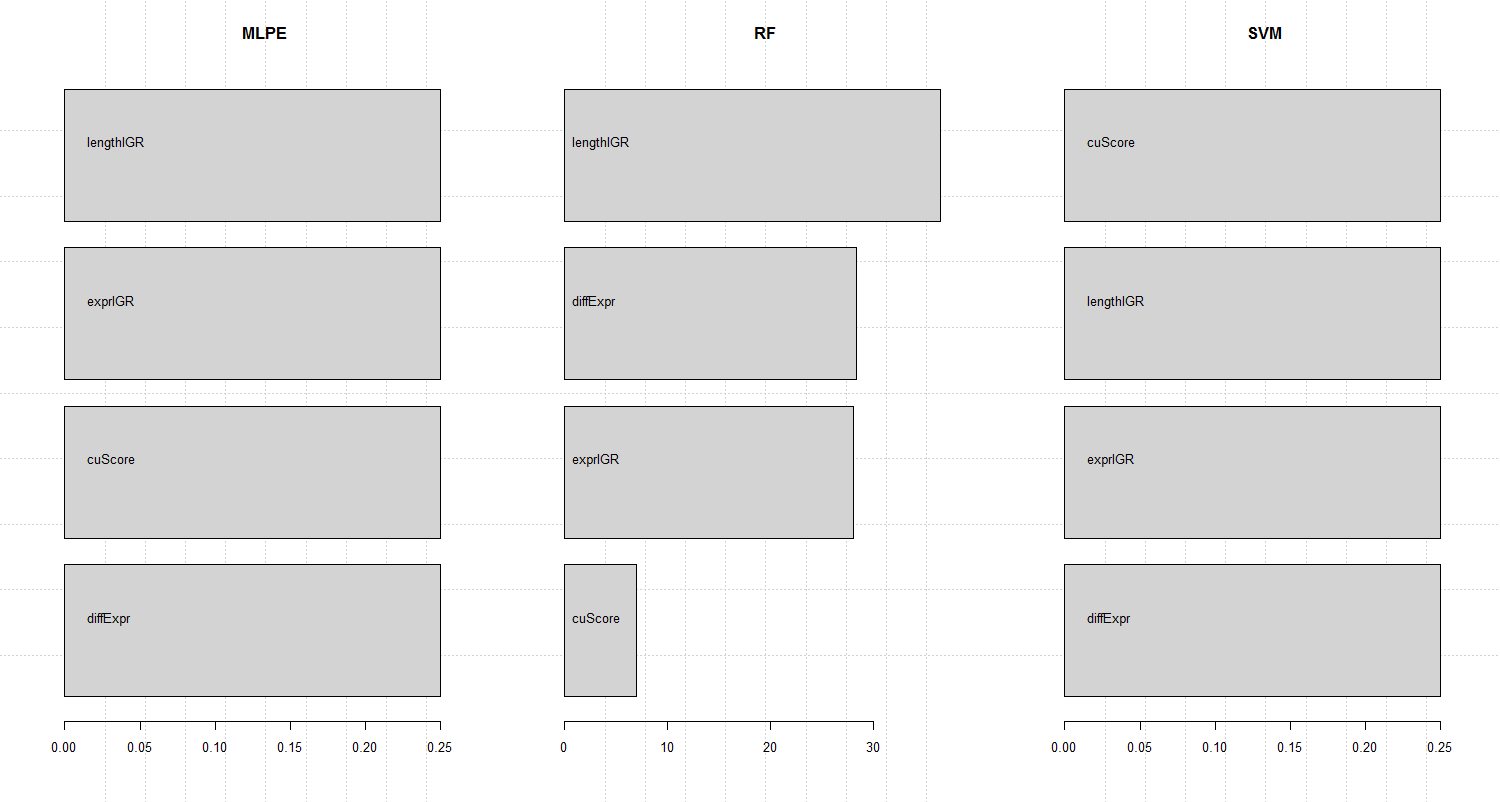


Figure 18 - Bar plots with the 1-D input importance”PG3”.

**Cross-validation**

**NN**

**AUC**

0.9741185 0.9774944 0.9966242 0.9992498 0.9789947 0.9774944 0.9791823 0.9797449 0.9969992 0.9988747

- M: 0.985877719429857
- SD: 0.0105175183768685

**ACC**

97.4359 99.1453 96.5812 97.4359 97.4359 97.4359 96.5812 97.4359 98.2906 99.1453

- M: 97.6923076923077
- SD: 0.905427269633661

pred

target NOP OP

NOP 286 24

OP 3 857

- TPR: 0.996511627906977
- Precision: 0.972758229284904
- FPR: 0.888888888888889
- ACC: 0.976923076923077
- ER: 0.0230769230769231

**RF**

**AUC**

0.9937304 0.9968652 1.0000000 0.9968652 1.0000000 1.0000000 1.0000000 1.0000000 1.0000000 1.0000000

- M: 0.998746081504702
- SD: 0.00219186802125425

**ACC**

97.5 97.5 100.0 95.0 95.0 95.0 100.0 95.0 95.0 100.0

- M: 97
- SD: 2.2973414586817

pred

target NOP OP

NOP 101 9

OP 3 287

- TPR: 0.989655172413793
- Precision: 0.969594594594595
- FPR: 0.75
- ACC: 0.97
- ER: 0.03

**SVM**

**AUC**

0.9516129 0.9722431 0.9516129 0.9561140 0.9516129 0.9296699 0.9457989 0.9677419 0.9677419 0.9516129

- M: 0.95457614403601
- SD: 0.0124438454654059

**ACC**

97.4359 97.4359 97.4359 96.5812 97.4359 95.7265 96.5812 98.2906 98.2906 97.4359

- M: 97.2649572649573
- SD: 0.785415883309985

pred

target NOP OP

NOP 284 26

OP 6 854

- TPR: 0.993023255813953
- Precision: 0.970454545454545
- FPR: 0.8125
- ACC: 0.972649572649573
- ER: 0.0273504273504274

**TEST-set**

**NN**

- AUC: 0.9614662
- ACC: 0.98076923076923
- ER: 0.0192307692307692

**RF**

- AUC: 1
- ACC: 0.98076923076923
- ER: 0.0192307692307692

**SVM**

- AUC: 0.8890977
- ACC: 0.903846153846154
- ER: 0.0961538461538462

PG3 – Genomic properties


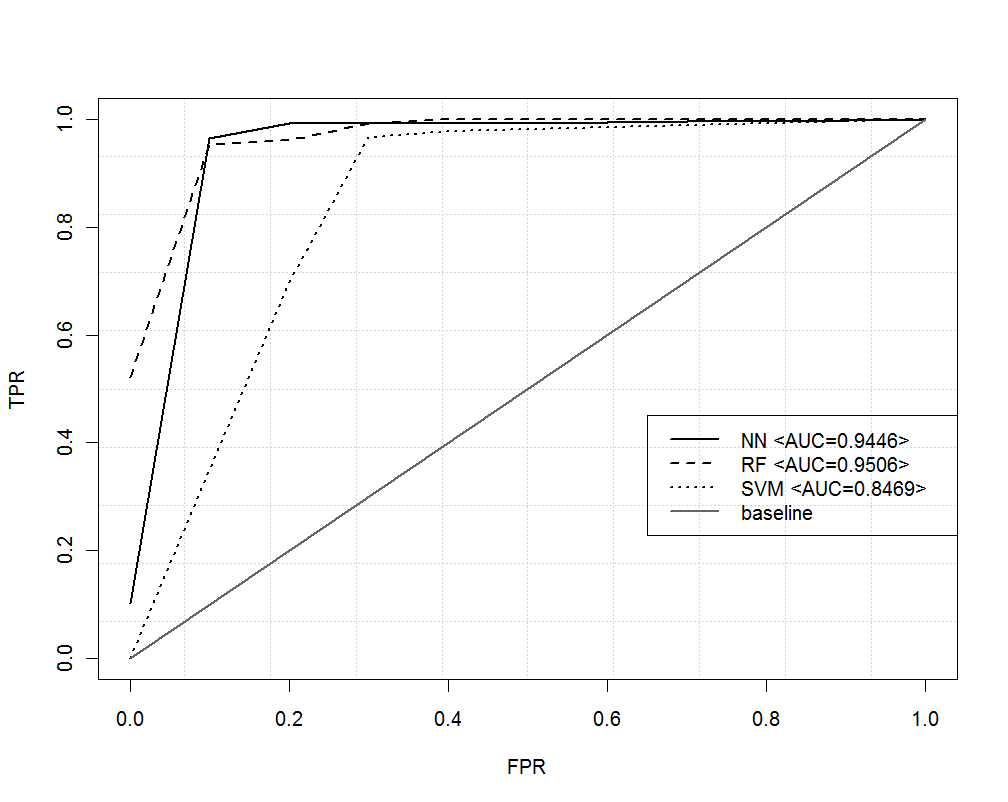


**Figure 19 – Roc curves achieved with the two genomic features (PG3).**

**Cross-validation**

**NN**

**AUC**

0.9424231 0.9257314 0.9583646 0.9771193 0.9379220 0.9681170 0.9336084 0.9191673 0.9534884 0.9304201

- M: 0.94463615903976
- SD: 0.0190119062352664

**ACC**

95.7265 94.8718 95.7265 94.0171 95.7265 95.7265 94.8718 94.8718 94.0171 95.7265

- M: 95.1282051282051
- SD: 0.703651796879113

pred

target NOP OP

NOP 273 37

OP 20 840

- TPR: 0.976744186046512
- Precision: 0.957810718358039
- FPR: 0.649122807017544
- ACC: 0.951282051282051
- ER: 0.0487179487179487

**RF**

**AUC**

0.9310345 1.0000000 0.9592476 0.9200627 0.9811912 0.9341693 0.9561129 0.9467085 0.8902821 0.9874608

- M: 0.950626959247649
- SD: 0.0334643475024031

**ACC**

97.5 100.0 97.5 97.5 92.5 95.0 92.5 92.5 90.0 92.5

- M: 94.75
- SD: 3.2167098442698

pred

target NOP OP

NOP 96 14

OP 7 283

- TPR: 0.975862068965517
- Precision: 0.952861952861953
- FPR: 0.666666666666667
- ACC: 0.9475
- ER: 0.0525

**SVM**

**AUC**

0.8432108 0.8373968 0.8651538 0.8593398 0.8432108 0.8593398 0.8373968 0.8593398 0.8373968 0.8270818

- M: 0.84688672168042
- SD: 0.0128597551032213

**ACC**

90.59829 89.74359 92.30769 91.45299 90.59829 91.45299 89.74359 91.45299 89.74359 89.74359

- M: 90.6837606837607
- SD: 0.94060250821762

pred

target NOP OP

NOP 223 87

OP 22 838

- TPR: 0.974418604651163
- Precision: 0.905945945945946
- FPR: 0.798165137614679
- ACC: 0.906837606837607
- ER: 0.0931623931623932

**TEST-set**

**NN**

- AUC: 1
- ACC: 0.961538461538462
- ER: 0.0384615384615385

**RF**

- AUC: 0.9962406
- ACC: 0.98076923076923
- ER: 0.0192307692307692

**SVM**

- AUC: 1
- ACC: 1
- ER: 0

PG3 – Transcriptomic properties


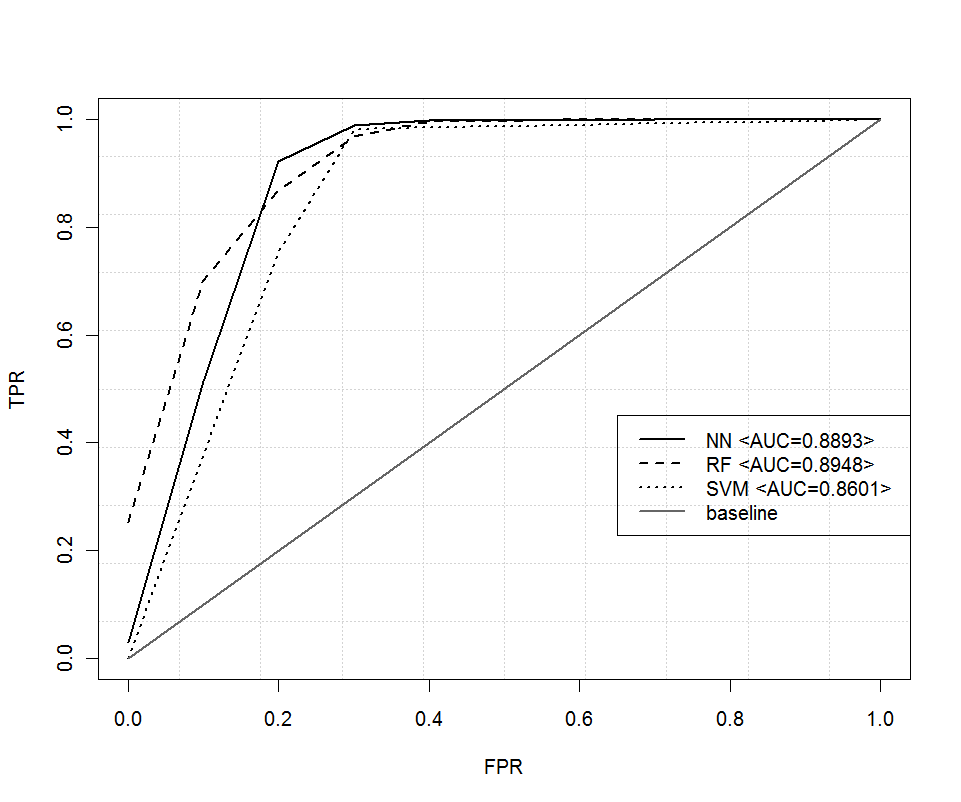


**Figure 20 – Roc curves achieved with the two transcriptomic features (PG3).**

**Cross-validation**

**NN**

**AUC**

0.9092273 0.9006002 0.9045386 0.8752813 0.8715304 0.8803451 0.8709677 0.8812828 0.9129782 0.8867217

- M: 0.889347336834209
- SD: 0.016045461580385

**ACC**

92.30769 92.30769 88.88889 90.59829 92.30769 91.45299 91.45299 89.74359 92.30769 92.30769

- M: 91.3675213675214
- SD: 1.23857920907602

pred

target NOP OP

NOP 235 75

OP 26 834

- TPR: 0.969767441860465
- Precision: 0.917491749174917
- FPR: 0.742574257425743
- ACC: 0.913675213675214
- ER: 0.0863247863247863

**RF**

**AUC**

0.9388715 0.9435737 0.9843260 0.9122257 0.8385580 0.8683386 0.8589342 0.9106583 0.8636364 0.8291536

- M: 0.894827586206897
- SD: 0.050904657475883

**ACC**

92.5 95.0 92.5 95.0 87.5 87.5 87.5 92.5 80.0 87.5

- M: 89.75
- SD: 4.63231403656243

pred

target NOP OP

NOP 81 29

OP 12 278

- TPR: 0.958620689655172
- Precision: 0.905537459283388
- FPR: 0.707317073170732
- ACC: 0.8975
- ER: 0.1025

**SVM**

**AUC**

0.8593398 0.8709677 0.8651538 0.8418980 0.8548387 0.8593398 0.8696549 0.8593398 0.8709677 0.8490248

- M: 0.860052513128282
- SD: 0.00964030728004853

**ACC**

91.45299 93.16239 92.30769 88.88889 92.30769 91.45299 91.45299 91.45299 93.16239 91.45299

- M: 91.7094017094017
- SD: 1.21208247214716

pred

target NOP OP

NOP 229 81

OP 16 844

- TPR: 0.981395348837209
- Precision: 0.912432432432432
- FPR: 0.835051546391753
- ACC: 0.917094017094017
- ER: 0.0829059829059829

**TEST-set**

**NN**

- AUC: 0.9605263
- ACC: 0.884615384615385
- ER: 0.115384615384615

**RF**

- AUC: 0.8242481
- ACC: 0.865384615384615
- ER: 0.134615384615385

**SVM**

- AUC: 0.8082707
- ACC: 0.884615384615385
- ER: 0.115384615384615

PG3 – Genomic properties + ExprIGR


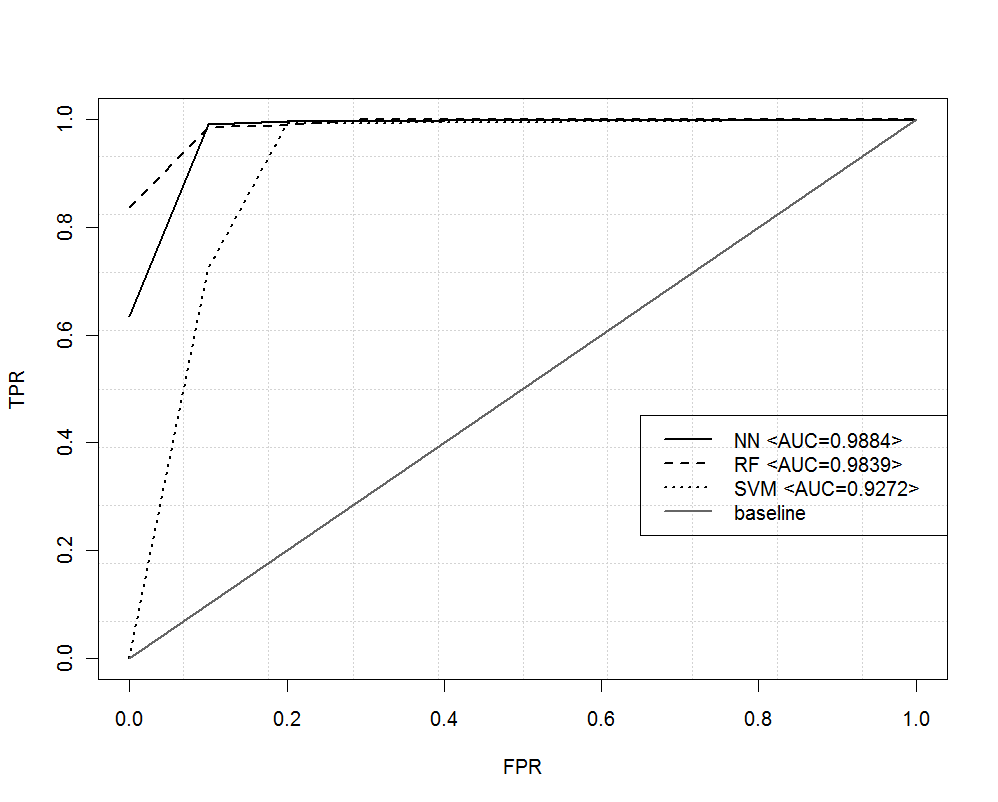


**Figure 21 – Roc curves achieved combining the first transcriptomic feature with the two genomic features (PG3).**

**Cross-validation**

**NN**

**AUC**

0.9795574 0.9921230 0.9863091 0.9973743 0.9883721 0.9797449 0.9893098 0.9797449 0.9984996 0.9932483

- M: 0.988428357089272
- SD: 0.00709548257096304

**ACC**

97.4359 95.7265 96.5812 98.2906 95.7265 98.2906 96.5812 96.5812 96.5812 96.5812

- M: 96.8376068376068
- SD: 0.90542726963366

pred

target NOP OP

NOP 290 20

OP 17 843

- TPR: 0.980232558139535
- Precision: 0.976825028968714
- FPR: 0.540540540540541
- ACC: 0.968376068376068
- ER: 0.0316239316239316

**RF**

**AUC**

0.9937304 0.9702194 1.0000000 0.9937304 0.9968652 1.0000000 1.0000000 0.9843260 0.9090909 0.9905956

- M: 0.98385579937304
- SD: 0.0278239533485939

**ACC**

95.0 92.5 100.0 95.0 95.0 97.5 97.5 95.0 95.0 92.5

- M: 95.5
- SD: 2.2973414586817

pred

target NOP OP

NOP 99 11

OP 7 283

- TPR: 0.975862068965517
- Precision: 0.962585034013605
- FPR: 0.611111111111111
- ACC: 0.955
- ER: 0.045

**SVM**

**AUC**

0.9354839 0.9354839 0.9238560 0.9296699 0.9354839 0.9135409 0.9354839 0.9354839 0.9193548 0.9077269

- M: 0.9271567891973
- SD: 0.0104758735962595

**ACC**

96.5812 96.5812 94.8718 95.7265 96.5812 94.8718 96.5812 96.5812 95.7265 94.0171

- M: 95.8119658119658
- SD: 0.940602508217614

pred

target NOP OP

NOP 267 43

OP 6 854

- TPR: 0.993023255813953
- Precision: 0.9520624303233
- FPR: 0.877551020408163
- ACC: 0.958119658119658
- ER: 0.0418803418803419

**TEST-set**

**NN**

- AUC: 0.9887218
- ACC: 0.961538461538462
- ER: 0.0384615384615385

**RF**

- AUC: 0.9962406
- ACC: 0.961538461538462
- ER: 0.0384615384615385

**SVM**

- AUC: 0.8928571
- ACC: 0.942307692307692
- ER: 0.0576923076923077

PG3 – Genomic properties + DiffExpr


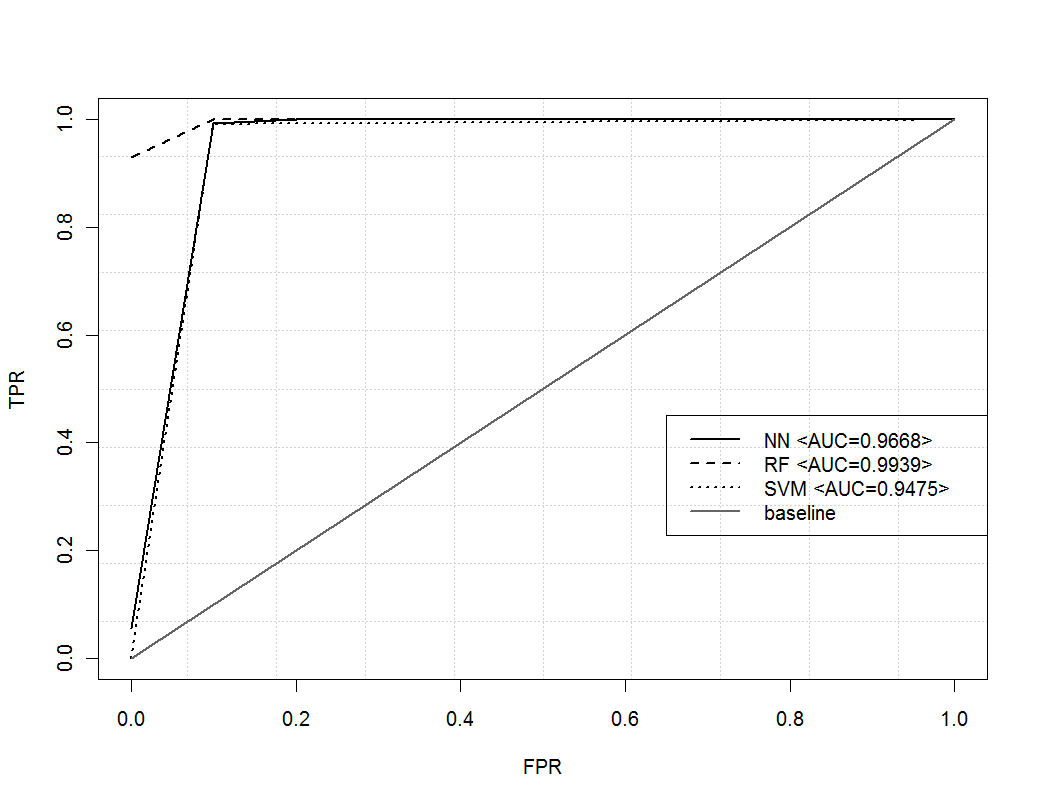


**Figure 22 – Roc curves achieved combining the second transcriptomic feature with the two genomic features (PG3).**

**Cross-validation**

**NN**

**AUC**

0.9561140 0.9731808 0.9761815 0.9679295 0.9752438 0.9555514 0.9583646 0.9673668 0.9608027 0.9776819

- M: 0.966841710427607
- SD: 0.00861437238386064

**ACC**

94.0171 97.4359 97.4359 97.4359 97.4359 96.5812 97.4359 96.5812 96.5812 95.7265

- M: 96.6666666666667
- SD: 1.09972986128882

pred

target NOP OP

NOP 281 29

OP 10 850

- TPR: 0.988372093023256
- Precision: 0.967007963594994
- FPR: 0.743589743589744
- ACC: 0.966666666666667
- ER: 0.0333333333333333

**RF**

**AUC**

0.9858934 1.0000000 0.9937304 1.0000000 0.9968652 0.9968652 1.0000000 1.0000000 0.9655172 1.0000000

- M: 0.993887147335423
- SD: 0.010920665098826

**ACC**

95.0 95.0 95.0 95.0 92.5 97.5 97.5 97.5 92.5 100.0

- M: 95.75
- SD: 2.37170824512628

pred

target NOP OP

NOP 93 17

OP 0 290

- TPR: 1
- Precision: 0.944625407166124
- FPR: 1
- ACC: 0.9575
- ER: 0.0425

**SVM**

**AUC**

0.9516129 0.9516129 0.9457989 0.9516129 0.9516129 0.9457989 0.9457989 0.9516129 0.9516129 0.9283571

- M: 0.947543135783946
- SD: 0.00727712533159636

**ACC**

97.4359 97.4359 96.5812 97.4359 97.4359 96.5812 96.5812 97.4359 97.4359 94.0171

M: 96.8376068376068"

SD: 1.06979962139707

pred

target NOP OP

NOP 280 30

OP 7 853

- TPR: 0.99186046511628
- Precision: 0.966024915062288
- FPR: 0.810810810810811
- ACC: 0.968376068376068
- ER: 0.0316239316239316

**TEST-set**

**NN**

- AUC: 0.994361
- ACC: 0.942307692307692
- ER: 0.057692307692307

**RF**

- AUC: 0.955827
- ACC: 0.903846153846154
- ER: 0.0961538461538462

**SVM**

- AUC: 0.8571429
- ACC: 0.923076923076923
- ER: 0.0769230769230769

HS2336 (All featutes)


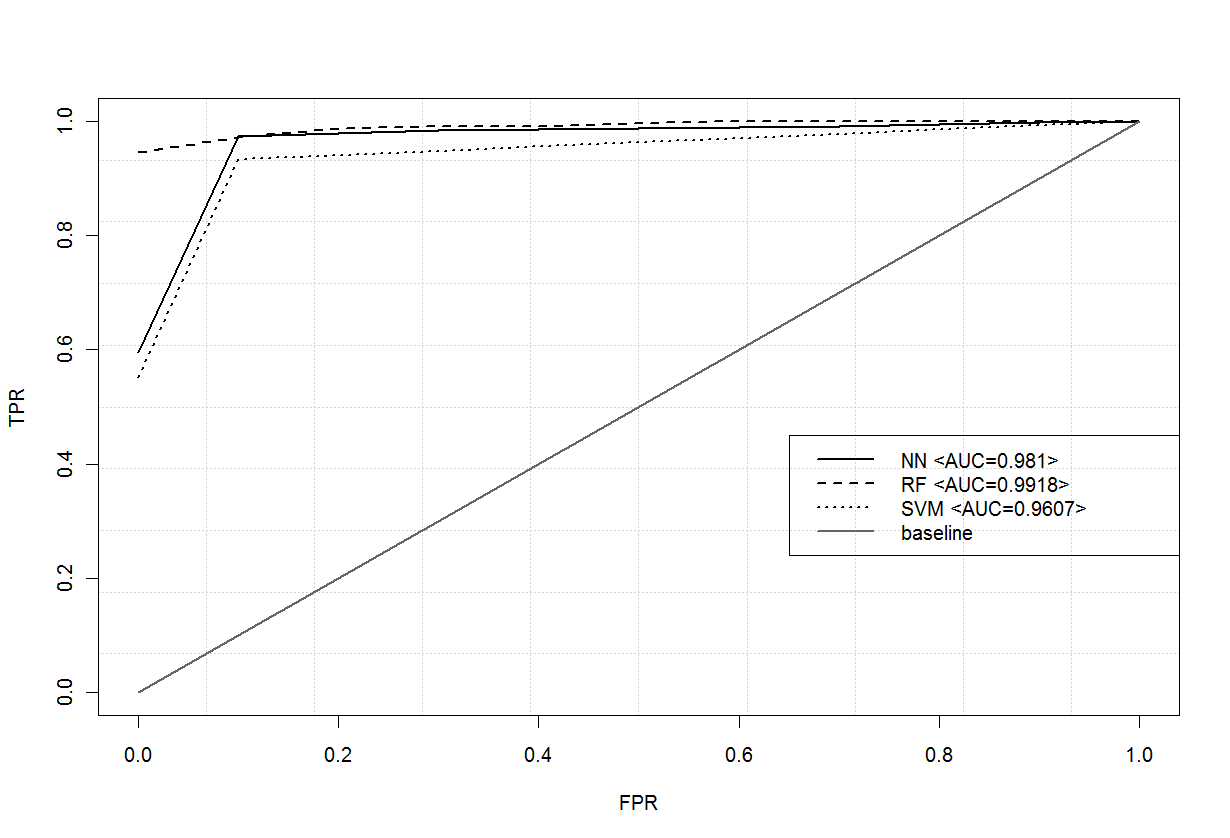


**Figure 23 – Roc curves for “HS2336”.**


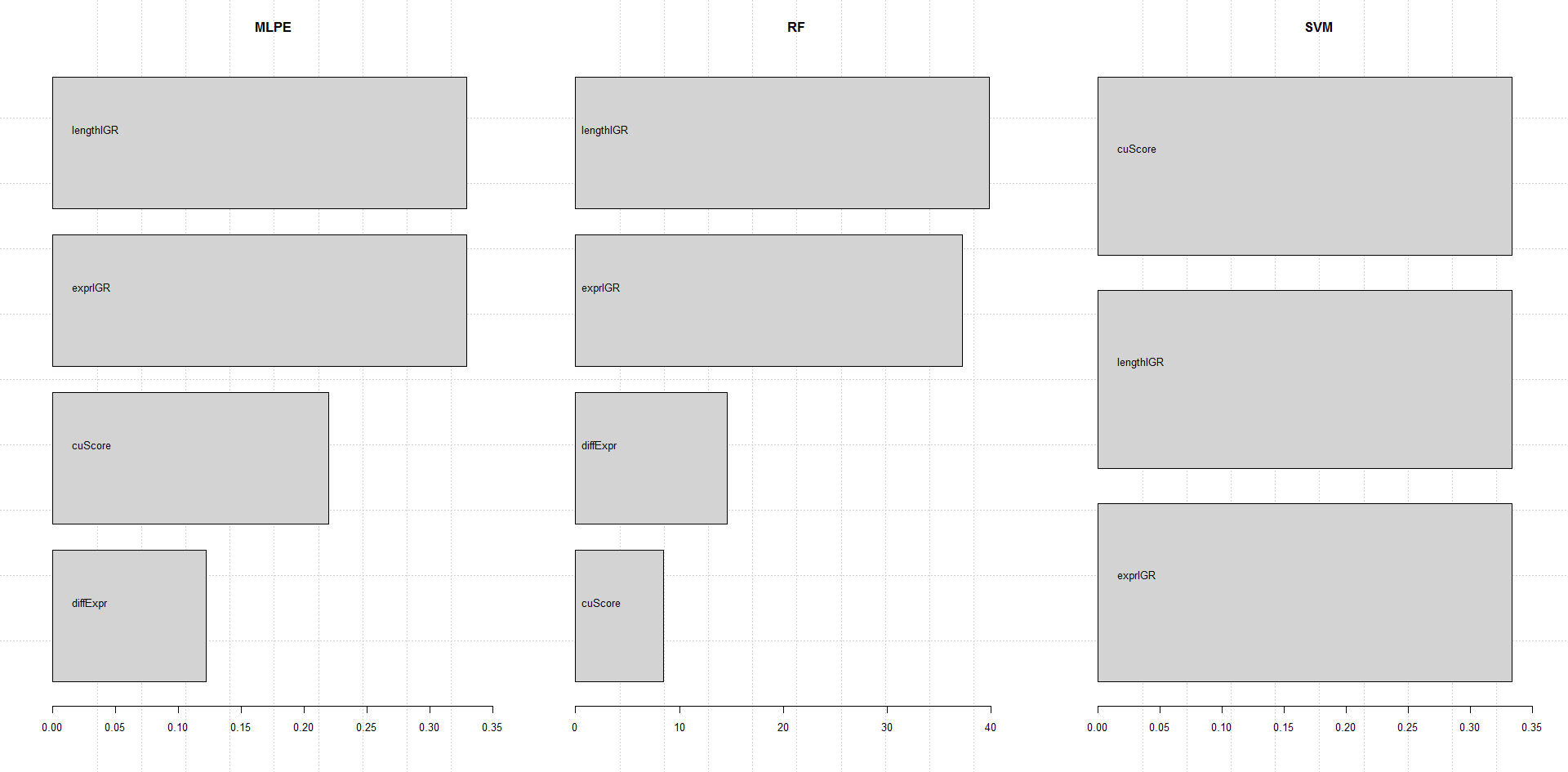


Figure 24 - Bar plots with the 1-D input importance”HS2336”.

**Cross-validation**

**NN**

**AUC**

0.9742369 0.9935897 0.9860195 0.9942613 0.9637363 0.9758852 0.9864469 0.9689866 0.9797314 0.9871795

- M: 0.981007326007326
- SD: 0.0102376359264981

**ACC**

95.72193 95.72193 95.72193 96.25668 93.58289 94.65241 97.32620 91.97861 94.11765 96.25668

- M: 95.1336898395722
- SD: 1.56314875353966

pred

target NOP OP

NOP 1120 50

OP 41 659

- TPR: 0.941428571428571
- Precision: 0.92947813822285
- FPR: 0.549450549450549
- ACC: 0.951336898395722
- ER: 0.0486631016042781

**RF**

**AUC**

0.9946581 0.9967949 0.9946581 0.9700855 0.9994658 0.9967949 1.0000000 0.9903846 0.9764957 0.9989316

- M: 0.991826923076923
- SD: 0.0102785514513549

**ACC**

96.8254 98.4127 96.8254 95.2381 95.2381 98.4127 98.4127 96.8254 96.8254 98.4127

- M: 97.1428571428571
- SD: 1.25208037737558

pred

target NOP OP

NOP 383 7

OP 11 229

- TPR: 0.954166666666667
- Precision: 0.970338983050847
- FPR: 0.388888888888889
- ACC: 0.971428571428571
- ER: 0.0285714285714286

**SVM**

**AUC**

0.9500000 0.9671551 0.9571429 0.9571429 0.9628816 0.9642857 0.9600122 0.9600122 0.9642857 0.9642857

- M: 0.96072039072039
- SD: 0.00500818920847578

**ACC**

96.25668 97.32620 96.79144 96.79144 96.79144 97.32620 96.79144 96.79144 97.32620 97.32620

- M: 96.951871657754
- SD: 0.360935057599227

pred

target NOP OP

NOP 1165 5

OP 52 648

- TPR: 0.925714285714286
- Precision: 0.992343032159265
- FPR: 0.087719298245614
- ACC: 0.96951871657754
- ER: 0.0304812834224599

**TEST-set**

**NN**

- AUC: 0.996205
- ACC: 0.97560975609756
- ER: 0.024390243902439

**RF**

- AUC: 1
- ACC: 0.98780487804878
- ER: 0.0121951219512195

**SVM**

- AUC: 0.957938
- ACC: 0.963414634146341
- ER: 0.0365853658536585

HS2336 – Genomic properties


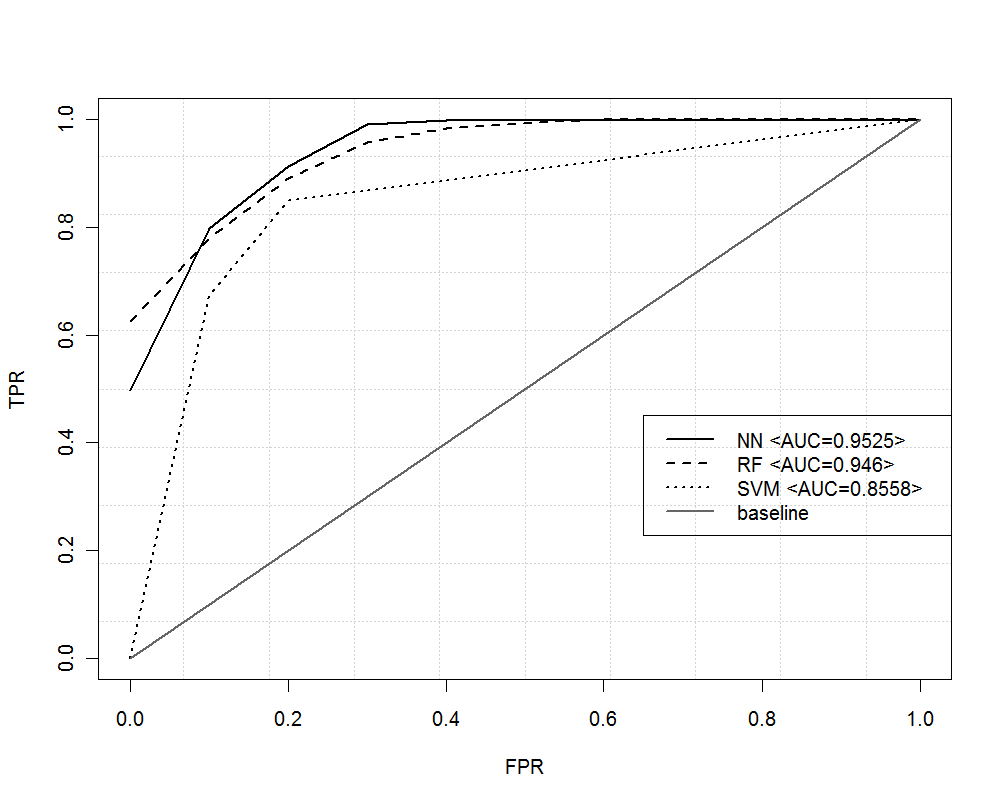


**Figure 25 – Roc curves achieved with the two genomic features (HS2336).**

**Cross-validation**

**NN**

**AUC**

0.9579976 0.9507937 0.9578755 0.9440171 0.9594628 0.9544567 0.9437118 0.9525031 0.9481074 0.9560440

- M: 0.952496947496947
- SD: 0.00572891566713693

**ACC**

86.63102 85.56150 87.16578 86.09626 87.16578 87.16578 85.56150 86.63102 85.02674 85.56150

- M: 86.2566844919786
- SD: 0.79916262999857

pred

target NOP OP

NOP 1056 114

OP 143 557

- TPR: 0.795714285714286
- Precision: 0.8301043219076
- FPR: 0.443579766536965
- ACC: 0.862566844919786
- ER: 0.137433155080214

**RF**

**AUC**

0.9444444 0.9636752 0.9471154 0.9615385 0.9636752 0.9497863 0.9220085 0.9172009 0.9487179 0.9417735

- M: 0.94599358974359
- SD: 0.0160364200849655

**ACC**

87.30159 84.12698 85.71429 88.88889 90.47619 87.30159 80.95238 84.12698 84.12698 85.71429

- M: 85.8730158730159
- SD: 2.74419100103491

pred

target NOP OP

NOP 344 46

OP 43 197

- TPR: 0.820833333333333
- Precision: 0.810699588477366
- FPR: 0.51685393258427
- ACC: 0.858730158730159
- ER: 0.141269841269841

**SVM**

**AUC**

0.8601954 0.8473138 0.8188034 0.8716117 0.8673382 0.8444444 0.8615995 0.8544567 0.8601343 0.8716117

- M: 0.855750915750916
- SD: 0.0159038188748381

**ACC**

86.09626 85.56150 82.35294 87.16578 86.63102 85.56150 86.63102 86.09626 87.16578 87.16578

- M: 86.0427807486631
- SD: 1.43601668885011

pred

target NOP OP

NOP 1023 147

OP 114 586

- TPR: 0.837142857142857
- Precision: 0.799454297407913
- FPR: 0.563218390804598
- ACC: 0.860427807486631
- ER: 0.139572192513369

**TEST-set**

**NN**

- AUC: 0.9810247
- ACC: 0.926829268292683
- ER: 0.073170731707317

**RF**

- AUC: 0.9652119
- ACC: 0.902439024390244
- ER: 0.097560975609756

**SVM**

- AUC: 0.8697027
- ACC: 0.853658536585366
- ER: 0.146341463414634

HS2336 – Transcriptomic properties


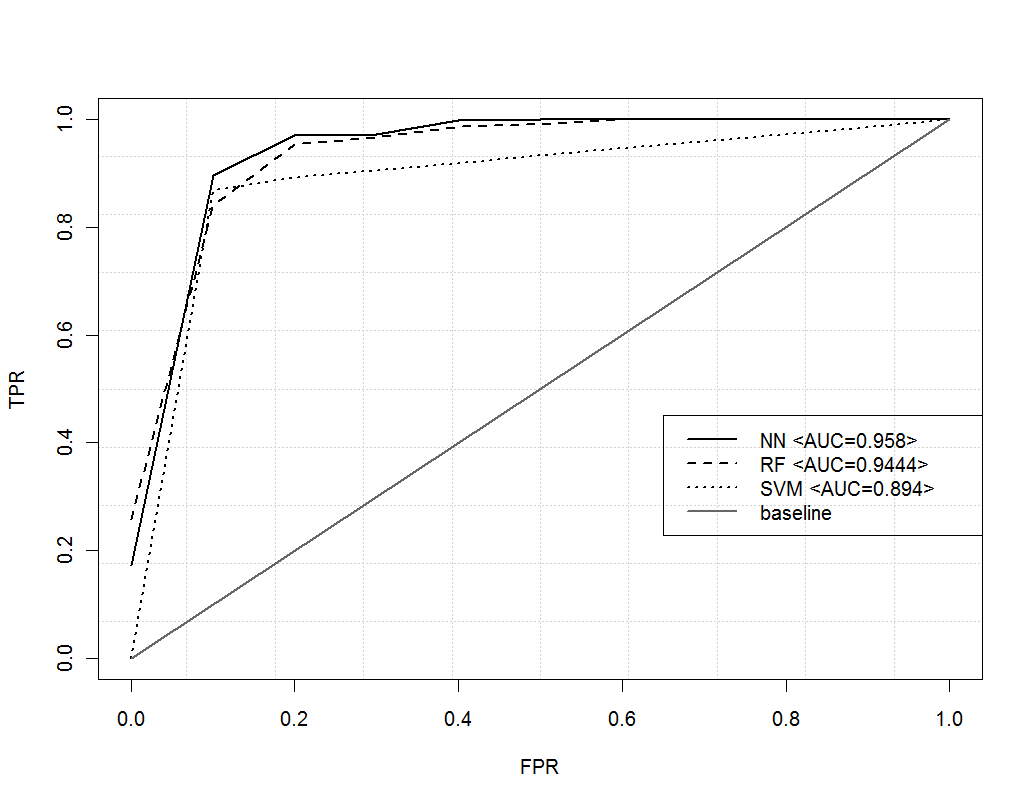


**Figure 26 – Roc curves achieved with the two transcriptomic features (HS2336).**

**Cross-validation**

**NN**

**AUC**

0.9594628 0.9554335 0.9590965 0.9578755 0.9573871 0.9592186 0.9577534 0.9565324 0.9589744 0.9578755

- M: 0.957960927960928
- SD: 0.00128968404486068

**ACC**

88.77005 88.77005 89.30481 89.30481 88.77005 88.77005 88.23529 88.77005 88.77005 88.77005

- M: 88.8235294117647
- SD: 0.303554124169814

pred

target NOP OP

NOP 1063 107

OP 102 598

- TPR: 0.854285714285714
- Precision: 0.84822695035461
- FPR: 0.511961722488038
- ACC: 0.888235294117647
- ER: 0.111764705882353

**RF**

**AUC**

0.9711538 0.9396368 0.9567308 0.9380342 0.9583333 0.9081197 0.9321581 0.9652778 0.9380342 0.9369658

- M: 0.944444444444444
- SD: 0.0186277732629943

**ACC**

90.47619 85.71429 88.88889 88.88889 85.71429 87.30159 88.88889 87.30159 90.47619 88.88889

- M: 88.2539682539683
- SD: 1.70629793614784

pred

target NOP OP

NOP 343 47

OP 27 213

- TPR: 0.8875
- Precision: 0.819230769230769
- FPR: 0.635135135135135
- ACC: 0.882539682539683
- ER: 0.117460317460317

**SVM**

**AUC**

0.8915751 0.9072650 0.8715507 0.8815629 0.8929792 0.8801587 0.9072650 0.8958486 0.9029915 0.9086691

- M: 0.893986568986569
- SD: 0.0129465559583507

**ACC**

89.30481 90.90909 88.23529 88.77005 89.83957 88.23529 90.90909 89.83957 90.37433 91.44385

- M: 89.7860962566845
- SD: 1.13998409663597

pred

target NOP OP

NOP 1064 106

OP 85 615

- TPR: 0.878571428571429
- Precision: 0.852981969486824
- FPR: 0.554973821989529
- ACC: 0.897860962566845
- ER: 0.102139037433155

**TEST-set**

**NN**

- AUC: 0.9354839
- ACC: 0.829268292682927
- ER: 0.170731707317073

**RF**

- AUC: 0.9038583
- ACC: 0.817073170731707
- ER: 0.182926829268293

**SVM**

- AUC: 0.8184693
- ACC: 0.829268292682927
- ER: 0.170731707317073

HS2336 – Genomic properties + ExprIGR


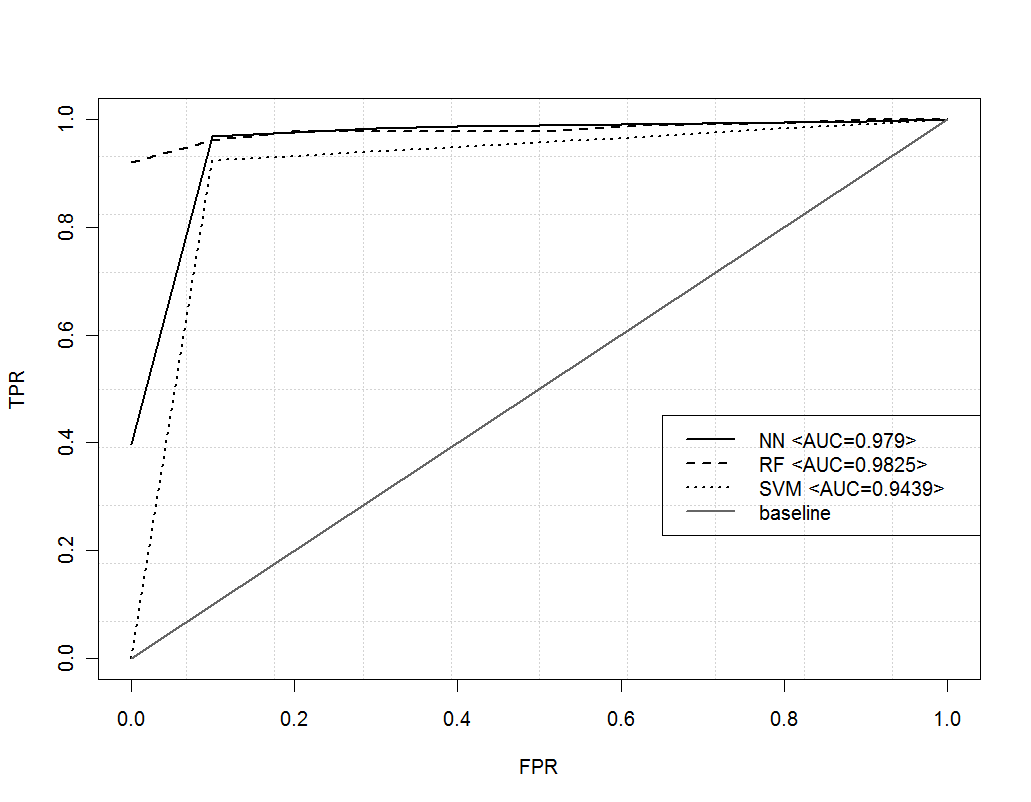


**Figure 27 – Roc curves achieved combining the first transcriptomic feature with the two genomic features (HS2336).**

**Cross-validation**

**NN**

**AUC**

0.9892552 0.9822955 0.9739927 0.9737485 0.9777778 0.9813797 0.9766789 0.9848596 0.9645299 0.9857753

- M: 0.979029304029304
- SD: 0.00723295091040687

**ACC**

93.58289 96.25668 95.18717 95.18717 94.65241 94.11765 94.65241 94.65241 92.51337 93.04813

- M: 94.385026737968
- SD: 1.10603151925052

pred

target NOP OP

NOP 1112 58

OP 47 653

- TPR: 0.932857142857143
- Precision: 0.918424753867792
- FPR: 0.552380952380952
- ACC: 0.94385026737968
- ER: 0.0561497326203209

**RF**

**AUC**

0.9695513 0.9967949 1.0000000 0.9925214 1.0000000 0.9658120 0.9941239 0.9743590 0.9679487 0.9642094

- M: 0.982532051282051
- SD: 0.0153155641801602

**ACC**

93.6508 96.8254 100.0000 95.2381 100.0000 96.8254 96.8254 96.8254 93.6508 95.2381

- M: 96.5079365079365
- SD: 2.21970126596858

pred

target NOP OP

NOP 385 5

OP 17 223

- TPR: 0.929166666666667
- Precision: 0.978070175438597
- FPR: 0.227272727272727
- ACC: 0.965079365079365
- ER: 0.0349206349206349

**SVM**

**AUC**

0.9485958 0.9514652 0.9329060 0.9443223 0.9471917 0.9429182 0.9300366 0.9429182 0.9628816 0.9357753

- M: 0.943901098901099
- SD: 0.00961246949068224

**ACC**

95.72193 95.72193 94.11765 95.18717 95.18717 94.65241 94.11765 94.65241 96.79144 94.11765

- M: 95.0267379679144
- SD: 0.87507577245159

pred

target NOP OP

NOP 1134 36

OP 57 643

- TPR: 0.918571428571429
- Precision: 0.94698085419735
- FPR: 0.387096774193548
- ACC: 0.950267379679144
- ER: 0.0497326203208556

**TEST-set**

**NN**

- AUC: 0.9993675
- ACC: 0.98780487804878
- ER: 0.0121951219512195

**RF**

- AUC: 0.99494
- ACC: 0.98780487804878
- ER: 0.0121951219512195

**SVM**

- AUC: 0.941809
- ACC: 0.951219512195122
- ER: 0.048780487804878

HS2336 – Genomic properties + DiffExpr


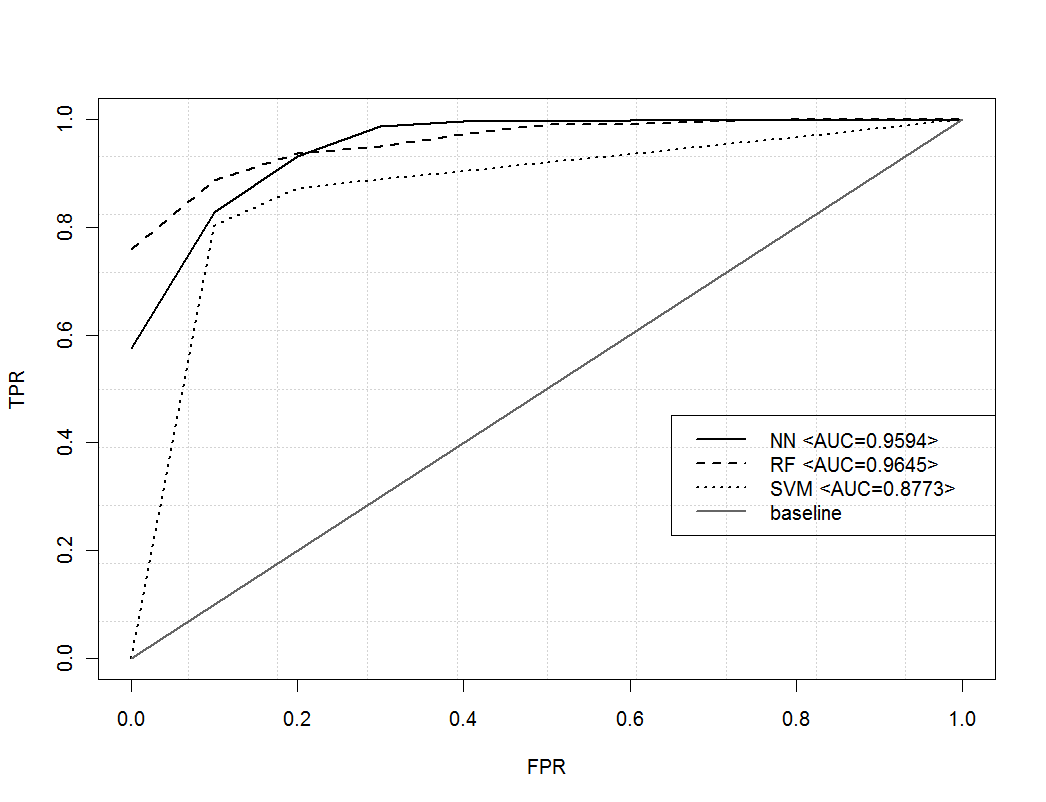


**Figure 28 – Roc curves achieved combining the second transcriptomic feature with the two genomic features (HS2336).**

**Cross-validation**

**NN**

**AUC**

0.9575092 0.9643468 0.9515263 0.9637363 0.9616606 0.9595849 0.9610501 0.9571429 0.9567766 0.9604396

- M: 0.95937728937729
- SD: 0.0037971414834784

**ACC**

88.23529 88.23529 86.63102 89.83957 88.77005 88.23529 88.77005 88.77005 87.16578 89.83957

- M: 88.4491978609626
- SD: 1.01463454336953

pred

target NOP OP

NOP 1078 92

OP 124 576

- TPR: 0.822857142857143
- Precision: 0.862275449101796
- FPR: 0.425925925925926
- ACC: 0.884491978609626
- ER: 0.115508021390374

**RF**

**AUC**

0.9882479 0.9786325 0.9722222 0.9716880 0.9786325 0.9423077 0.9807692 0.9652778 0.9294872 0.9375000

- M: 0.964476495726496
- SD: 0.0205139488303563

**ACC**

90.47619 88.88889 90.47619 87.30159 92.06349 87.30159 93.65079 92.06349 79.36508 88.88889

- M: 89.047619047619
- SD: 3.99111580225314

pred

target NOP OP

NOP 359 31

OP 38 202

- TPR: 0.841666666666667
- Precision: 0.866952789699571
- FPR: 0.449275362318841
- ACC: 0.89047619047619
- ER: 0.10952380952381

**SVM**

**AUC**

0.8701465 0.8929792 0.8615995 0.8972527 0.8772894 0.8929792 0.8844322 0.8601954 0.8558608 0.8801587

- M: 0.877289377289377
- SD: 0.0149023297674774

**ACC**

87.70053 89.83957 86.63102 90.37433 88.23529 89.83957 88.77005 86.09626 86.63102 88.23529

- M: 88.235294117647
- SD: 1.49137259631743

pred

target NOP OP

NOP 1050 120

OP 100 600

- TPR: 0.857142857142857
- Precision: 0.833333333333333
- FPR: 0.545454545454545
- ACC: 0.88235294117647
- ER: 0.117647058823529

**TEST-set**

**NN**

- AUC: 0.9607843
- ACC: 0.878048780487805
- ER: 0.121951219512195

**RF**

- AUC: 0.9611006
- ACC: 0.878048780487805
- ER: 0.121951219512195

**SVM**

- AUC: 0.9025933
- ACC: 0.902439024390244
- ER: 0.097560975609756
